# Genetic basis and evolution of rapid cycling in railway populations of tetraploid *Arabidopsis arenosa*

**DOI:** 10.1371/journal.pgen.1007510

**Published:** 2018-07-05

**Authors:** Pierre Baduel, Ben Hunter, Sarang Yeola, Kirsten Bomblies

**Affiliations:** 1 Department of Organismic and Evolutionary Biology, Harvard University, Cambridge, MA, United States of America; 2 École des Mines de Paris, Paris, France; 3 University of Maryland, Baltimore, MD, United States of America; The University of North Carolina at Chapel Hill, UNITED STATES

## Abstract

Spatially structured plant populations with diverse adaptations provide powerful models to investigate evolution. Human-generated ruderal habitats are abundant and low-competition, but are challenging for plants not adapted to them. Ruderal habitats also sometimes form networked corridors (e.g. roadsides and railways) that allow rapid long-distance spread of successfully adapted variants. Here we use transcriptomic and genomic analyses, coupled with genetic mapping and transgenic follow-up, to understand the evolution of rapid cycling during adaptation to railway sites in autotetraploid *Arabidopsis arenosa*. We focus mostly on a hybrid population that is likely a secondary colonist of a railway site. These mountain railway plants are phenotypically similar to their cosmopolitan cousins. We thus hypothesized that colonization primarily involved the flow of adaptive alleles from the cosmopolitan railway variant. But our data shows that it is not that simple: while there is evidence of selection having acted on introgressed alleles, selection also acted on rare standing variation, and new mutations may also contribute. Among the genes we show have allelic divergence with functional relevance to flowering time are known regulators of flowering, including *FLC* and *CONSTANS*. Prior implications of these genes in weediness and rapid cycling supports the idea that these are “evolutionary hotspots” for these traits. We also find that one of two alleles of *CONSTANS* under selection in the secondary colonist was selected from rare standing variation in mountain populations, while the other was introgressed from the cosmopolitan railway populations. The latter allele likely arose in diploid populations over 700km away, highlighting how ruderal populations could act as allele conduits and thus influence local adaptation.

## Author summary

To fully understand evolution and adaptation we not only need to understand the mechanisms underlying the evolution of novel traits, but also the sources and history of adaptive alleles. Plants adapted to human-generated (ruderal) habitats provide excellent models for rapid adaptation. Some ruderal habitats also form corridors–e.g. railways or roadsides–that could help spread the alleles of adapted variants widely. Here we study adaptation to railways in *Arabidopsis arenosa*, both of a cosmopolitan railway variant and a secondary colonist. To study the mechanisms of adaptation, we use a variety of approaches, ranging from whole genome analyses, to functional dissection of alleles conferring weedy phenotypes to railway plants,. We find that selection in the secondary colonist acted not only on alleles brought in by gene flow from the widely distributed railway type, but also on rare standing variation and new mutations. Among the genes involved are known regulators of flowering time, including one that is considered an “evolutionary hotspot” for switches to weediness and rapid cycling. Strikingly, one selected allele in the secondary colonist arose in populations over 700km away and arrived via the cosmopolitan railway variant, highlighting how such ruderal populations can act as allele conduits and can influence adaptation.

## Introduction

Human-associated ruderal sites, such as railways, roadsides and field margins are relatively recent and challenging habitats for plants [1–7]. Such ruderal sites serve as powerful model systems for adaptation. But ruderal adaptation, once attained, can provide new opportunities and can also change the spatial structure of a species: Ruderal sites are often low competition and abundant, while human-generated “corridor” habitats like railways and roadsides can facilitate long-distance dispersal of adapted genotypes (e.g. [8–11]). These corridors can also allow colonists to come into contact with, and perhaps hybridize with, related species or populations with different adaptations they would otherwise have been isolated from [12].

Colonists of ruderal sites have several clear phenotypic features that often distinguish them from their non-ruderal counterparts. Ruderal plants must withstand or evade a variety of stresses including high light, temperature fluctuations, or late summer droughts. An important additional factor on railways may also be that rail beds are cleared of plant life in summers, often annually. For example, since about 1920 German railway ballast has been regularly subjected to thermal treatments and since the 1960’s herbicide applications at a rate six times that used in agricultural settings [13]. Such lethal factors provide truncation selection, which can drive rapid trait evolution [14] and has been suggested as a driver of repeated evolution of rapid cycling in plants in marginal habitats (e.g. [1,15]), since rapid cycling, often coupled with loss of perenniality, is a mechanism by which plants can avoid seasonal stresses (e.g. [1–5,7,16]).

Here we study the genetic basis and biogeographic context of the evolution of rapid cycling in railway colonists of the otherwise non-ruderal *Arabidopsis arenosa*. This species is a close relative of *A*. *thaliana* [17,18] that exists in both diploid and autotetraploid forms [19]. All *A*. *arenosa* populations, including ruderal variants, remain obligate outcrossers [20]. Most diploid and autotetraploid populations of *A*. *arenosa* are perennial and found on sheltered rock outcrops or slopes usually in forests or on mountains, but within the autotetraploids, one genetic lineage colonized lowland ruderal sites and is now widely distributed across the railways of central and northern Europe [21]. All railway plants tested to date are rapid cycling and lack a vernalization response (need for winter cold exposure), in contrast to their relatives in mountain sites, which are all perennial and late flowering in the lab [22], albeit to varying extents. We have also previously shown that ruderal *A*. *arenosa* plants are constitutively heat and cold stress tolerant [22]. The adaptations seen in ruderal *A*. *arenosa* are typical of plants found in such marginal habitats.

In the evolution literature, it has been suggested that genetic isolation by environment is generally more prevalent than genetic isolation by geographic distance [23]. This is evident in *Arabidopsis arenosa*: geographically widely dispersed ruderal populations found on railways are genetically very similar, yet remain clearly distinct from geographically proximal populations found in different habitats, e.g. rock outcrops in forests [21]. We also know that there is inter-ploidy gene flow, and perhaps also inter-habitat gene flow within this system [21]. Thus *Arabidopsis arenosa* provides an opportunity to study the roles of alleles of different histories during adaptation.

Here we primarily focus on understanding the evolution of early flowering in an autotetraploid population we identified previously on a railway site in Berchtesgaden (BGS) in the Bavarian Alps [21]. We use whole-genome resequencing, transcriptome analysis, genetic mapping, transgenics, and phylogeographic analyses to begin unravelling the complex history of the evolution of rapid cycling in this population.

## Results

### BGS, a gene expression outlier among *A*. *arenosa* railway populations

**Fig 1:**
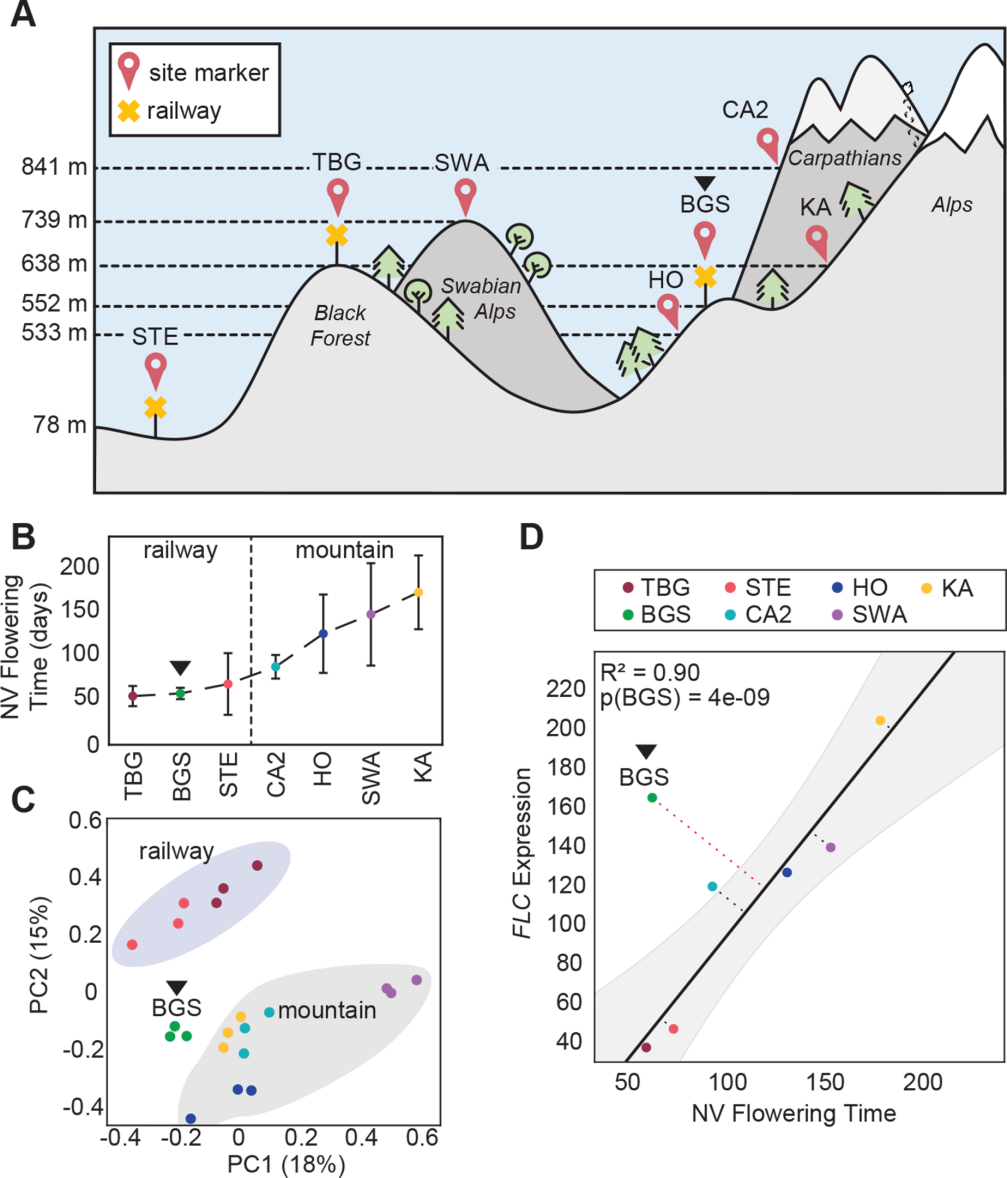
BGS, a transcriptomic outlier among railway populations. (A) Schematic representation of the habitats and altitudes where the railway populations (yellow cross markers): TBG, STE, and BGS (black triangle across all subfigures) and mountain populations: SWA, KA, HO, CA2 were sampled (GPS coordinates in S1 Table). (B) Vernalization response of populations reproduced from Baduel et al. as the difference between non-vernalized and vernalized flowering time. All railway populations present almost identical null vernalization responses. (C) First two principal components (PC1 and 2 with percentage of variance explained) of Principal Component Analysis (PCA) of the expression profiles of the 500-most variable genes. Railway and mountain populations group closely together by site-type. (D) Correlation analysis between *FLC* average expression and average non-vernalized (NV) flowering time. The linear regression model after exclusion of BGS is plotted in solid black. Grey area represents the 95% predicted confidence intervals around regression line. Dotted lines are the residual (orthogonal distance) for each data point from the regression line. The p-value for the likelihood to obtain a residual as observed with BGS from residual distribution is indicated as p(BGS).

To study the mechanism(s) of rapid cycling in autotetraploid *A*. *arenosa*, we first sought to identify genes whose expression is correlated with one of the most tractable traits associated with weediness, flowering time. To do this, we grew plants in controlled conditions from seeds collected from three exposed railway sites (TBG, STE, and BGS) and four sheltered hill / mountain populations (SWA, HO, KA, CA2; Fig 1A and S1 Table). We previously showed that in laboratory conditions, railway plants are almost all early flowering and not responsive to vernalization (prolonged cold treatment), while mountain plants show wider variation, but are consistently later flowering and respond strongly to vernalization [22]. For clarity these data are shown again here (Fig 1B). We quantified gene expression in leaves of three 3-week-old un-vernalized individuals from each population using read counts from whole-transcriptome sequencing (RNA-seq) aligned to the closely related *A*. *lyrata* reference genome [24]. Principal Component Analysis (PCA) of the genome-wide transcriptional profiles groups early flowering plants from geographically distant railway populations TBG (SW Germany) and STE (Central Poland) together. However, the equally early BGS railway plants from the Alps (SE Germany) grouped more closely with late flowering mountain populations, albeit in an intermediate position (Fig 1C). This is consistent with our previous finding that BGS is also genetically intermediate [21]. We thus refer to BGS as a “mountain railway” population to distinguish it from the other more widespread “lowland railway” populations (represented here by TBG and STE).

By analyzing transcriptomes of all phenotyped plants, we identified 76 genes whose expression correlated significantly with flowering time (S2 Table, see Methods). These flowering-correlated genes are functionally diverse and include only one known flowering time gene [25], the floral repressor *FLC*. Expression of *FLC* is also known to be strongly correlated with flowering time in wild accessions of *A*. *thaliana* [26–33] and here we also found a trend of low *FLC* expression in early-flowering and high *FLC* in late-flowering accessions. The two early flowering lowland railway populations (TBG and STE) have virtually undetectable *FLC* (Fig 1D), while late flowering mountain populations have high *FLC* expression, putting *FLC* among the 5% most strongly differentially expressed genes between railway and mountain accessions (S1A Fig). The mountain railway population BGS, however, did not follow this general trend: When excluding BGS, *FLC* expression levels were well correlated with flowering time (R^2^ = 0.90; Fig 1D), but though they flower as early as plants from STE and TBG (Fig 1B), BGS plants have expression levels of *FLC* as high as late flowering mountain populations (Fig 1D). Many other genes among the 76 flowering-correlated genes show a similar trend: BGS shows expression levels characteristic of early flowering plants for only 18 of the 76 otherwise flowering-correlated genes (S2 Table).

**Fig 2:**
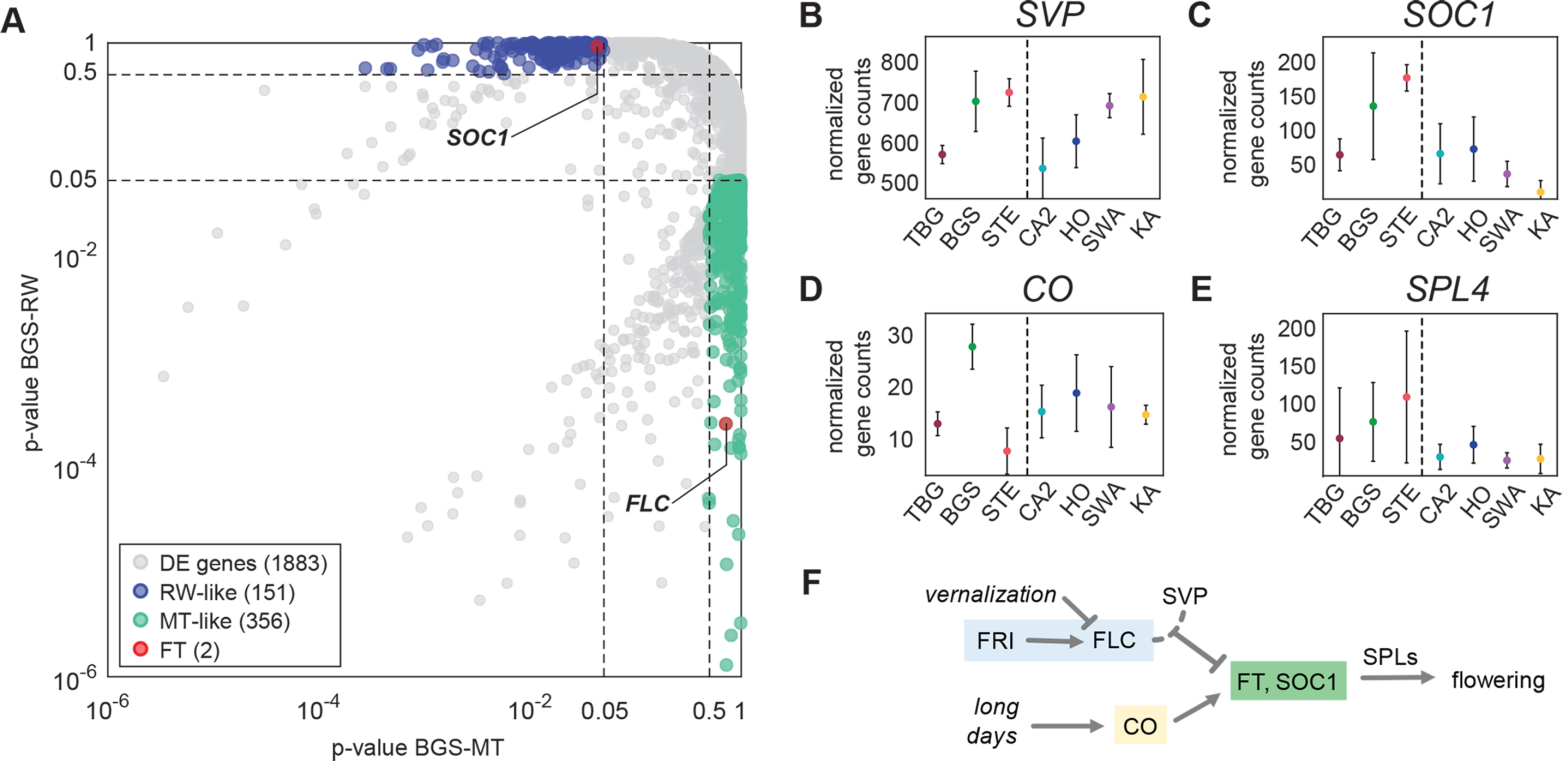
Gene expression patterns in BGS. (A) Railway (RW) and mountain (MT) like expression patterns in BGS measured by BGS-MT (x-axis) and BGS-RW (y-axis) comparisons among genes differentially expressed (DE) between mountain and lowland railway populations. (B, C, D, E) Normalized expression levels of *SVP* (B), *SOC1* (C), *CO* (D), and *SPL4* (E) across populations (error bars: SD). (F) Schematic representation of the interaction between vernalization (blue) and photoperiod (yellow) pathways. On one side the vernalization pathway represses the expression of flowering activators *FT* and *SOC1* through the FLC-SVP complex, while on the other the photoperiod pathway integrator *CO* activates them. Among the cascade of downstream targets of *SOC1* and *CO* are *SPL* factors including, *SPL4* [74].

To more quantitatively assign genes as having railway-like or mountain-like expression in BGS, we performed a multiple comparison of gene expression levels between BGS, mountain and lowland railway populations using a post-hoc Tukey honest significance test of a one-way ANOVA (see Methods). With this approach, we identified 1883 differentially expressed (DE) genes between mountain and lowland railway populations (p_RW-MT_<0.05). Of these, 356 genes had mountain-like expression levels in BGS (p_BGS-RW_<0.05 and p_BGS-MT_>0.5) and 151 genes railway-like expression (p_BGS-RW_>0.5 and p_BGS-MT_<0.05, Fig 2A). This >2 fold tendency towards mountain-like expression confirms that gene expression levels of early flowering BGS are overall more similar to late-flowering mountain populations. The list of differentially expressed genes included two flowering-time genes (based on a previously published flowering-time gene list [25]): *FLC*, which had mountain-like (high) expression in BGS, and *SUPPRESSOR OF OVEREXPRESSION OF CONSTANS1 (SOC1)* which had railway-like (high) expression in BGS (Fig 2). *SOC1* promotes flowering, and is directly repressed by a complex of FLC and another protein, SVP in *A*. *thaliana* [34,35] (the gene encoding SVP, like *FLC*, is also highly expressed in BGS; Fig 2B). *SPL4*, a direct target of *SOC1* [36], is also expressed higher in BGS than in late flowering mountain plants (Fig 2E) suggesting the high *SOC1* expression is functionally relevant.

The elevated *SOC1* and *SPL4* expression in BGS is consistent with its early flowering, but its high expression of *FLC* is not. These trends suggest that in these plants either *FLC* is not effective in repressing *SOC1*, or that *SOC1* activation occurs despite high *FLC* activity (i.e. that *FLC* is active, but circumvented). A plausible candidate for such circumvention is *CONSTANS* (*CO*), which can directly activate *SOC1* even in the presence of high *FLC* levels [37,38] (Fig 2F). We indeed observed variation in *CO* expression levels among populations, with BGS having the highest levels, STE and TBG having consistently low expression, and mountain populations having a range of intermediate expression levels (Fig 2D).

### Genetic mapping of flowering time

To understand the genetic mechanisms underlying early flowering in BGS and compare this to the lowland railway populations, we took a genetic mapping approach. We grew 795 and 845 F_2_ plants derived from TBG x SWA and BGS x KA crosses, respectively (TBG is our earliest flowering lowland railway population while both SWA and KA are similarly late flowering mountain types). We quantified flowering time by measuring initiation of inflorescence outgrowth (bolting). The phenotype distribution of TBG x SWA F_2_ plants showed three modes, around 42, 55, and 68 days to bolting with a tail of plants that did not bolt before the end of the experiment at 80 days (approximately 1/8 of plants; S3A and S3B Fig). In contrast, the distribution of BGS x KA F_2_ plants was bi-modal with a single early-flowering mode around 50 days and a group of very late flowering individuals (~1/4 of plants) that had not bolted by 140 days.

**Fig 3:**
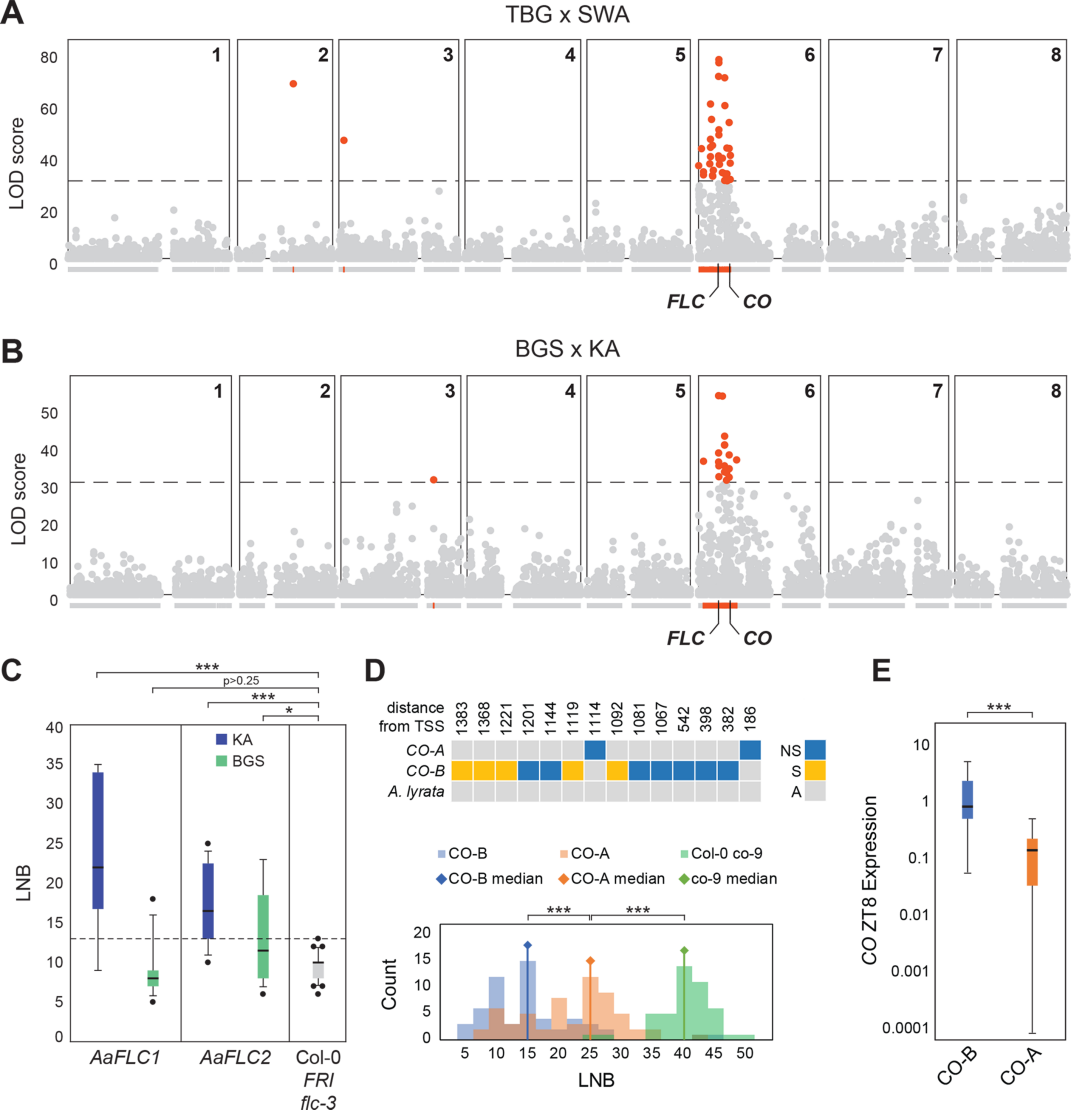
Phenotypic impact of *FLC* and *CO* in BGS. (A-B) BSA mapping of flowering time in TBG x SWA and BGS x KA F_2_s. Distribution of single-marker LOD scores for time to bolting across the 8 scaffolds in TBG x SWA (A) and BGS x KA F_2_s (B) above the gene models for each scaffold. High LOD (LOD>30) markers and genes within high-LOD regions are highlighted in red. (C) Boxplot of flowering time measured by leaf number at bolting (LNB) of Kanamycin-resistant T1s with 35S-driven cDNAs of both *AaFLC1* (blue) and *AaFLC2* (green) from KA (left panel) and BGS (middle panel). For comparison the flowering time of the FRI flc-3 Col-0 background line is plotted in the third panel in grey. The latest flowering time observed in the background line (LNB = 13) is represented by a dotted line. The numbers above each box indicate the correlations between LNB and *FLC* expression (S2 Fig). Two late-flowering transgenic individuals obtained with BGS *AaFLC1* that nevertheless have low FLC expression are indicated with black triangles. (D) Distribution of flowering time (LNB) of Kanamycin-resistant T1s with genomic constructs of *CO-A* (orange) and *CO-B* (blue) which differ by 14 SNPs within their CDS (upper panel) compared to co-9 mutants (green). The medians of each distribution are indicated by diamonds. (E) Normalized *CO* expression at ZT8 of *CO-A* (orange) and *CO-B* (blue) T1s. (* = p < 0.05, ** = p < 0.01; *** = p < 0.001).

We used restriction-associated reduced-representation sequencing (RADseq) to genotype 452 and 284 individuals selected from each mode in the two F_2_ populations derived from the TBG x SWA and BGS x KA crosses. We obtained 7907 and 8666 informative single nucleotide polymorphisms (SNPs) for the two F_2_ populations, respectively, after filtering for a minimum coverage of 40% of individuals per SNP. We tested for correlations of markers in each group with flowering time. In both populations, there was a very strong association with days to bolting in overlapping regions on the upper arm of scaffold 6 and nowhere else in the genome (Fig 3A and 3B). Regions of high LOD scores (>30, as maximum LOD scores measured over 500 random permutations were 28.44 and 27.92 respectively) in TBG x SWA and BGS x KA spanned 6.5 Mb and 6.8 Mb respectively, with a shift down the chromosome in BGS x KA (Fig 3A and 3B). The peaks are strongly significant in both F_2_ populations (p-values of marker with highest LOD: 2e-18 in TBG x SWA and 4e-13 in BGS x KA). Together the two intervals of high LOD in the two populations define a 7.8 Mb region hereafter referred to as FT-peak, which spans 1877 genes. In both TBG x SWA and BGS x KA the SNP of maximum LOD score falls close to *FLC* (within 135kb and 75kb respectively; Fig 3A and 3B), though other genes known to be associated with flowering time in *A*. *thaliana* are also found within the FT-peak region, including *MYB33* and *CO*.

Among the 5% most strongly differentially expressed genes between lowland railway and mountain populations (S1A and S1B Fig) that map within the FT-peak region, *FLC* was the only one among 174 *A*. *thaliana* genes previously associated with flowering time regulation [25]. Given its higher in BGS (Fig 2D), *CO* is also an intriguing candidate for controlling flowering time differences segregating in the BGS x KA F_2_ population. In TBG x SWA, *CO* is located at the very edge of the interval within 78kb of the most downstream SNP of LOD score above 30, but in BGS x KA *CO* is located over 1.3 Mb from the end of the high-LOD region (Fig 3A and 3B). We estimated individual effects of high LOD markers across the FT-peak region (see S1 Text, and S3C Fig) and this supported the potential involvement of both *FLC* and *CO* in early flowering in the BGS x KA cross.

Using informative RADseq markers across the mapping region in the BGS x KA cross, we were able to identify parental chromosomes and infer partial or complete genotypes for a subset of 135 individuals (S2 Text). Among these, we found that three of the four parental chromosomes segregating in the F_2_ that originate from BGS quantitatively confer early flowering. Both the *FLC* and *CO* regions contribute to this effect with a more pronounced contribution from *CO*. All four of the KA chromosomes, and one of the BGS chromosomes, are associated with delayed flowering, showing that late flowering alleles do still segregate in BGS. For two of the parental chromosomes (repressing and accelerating) that segregate in the F_2_, the effect is stronger at *CO* than at *FLC*, while for another, an effect is only seen at *FLC* (see S2 Text).

### Indications of *FLC* decay in BGS

*FLC* was an obvious candidate within the FT QTL region in both crosses, but its expression differs between the early flowering strains used (high in BGS, absent in TBG). Since high *FLC* expression is generally associated with late flowering in both *A*. *thaliana* [26], and *A*. *arenosa* (this study), we hypothesized that the expressed allele in BGS might be non-functional. In *A*. *arenosa* the *FLC* locus contains two full-length (*AaFLC1*, *AaFLC2*) and one truncated (*AaFLC3*) copy [39], so we first established which copies are expressed using an allele-specific RNAseq approach we previously used (Baduel et al. [22]). We found that 90% of *FLC* expression in BGS and all mountain populations was contributed by *AaFLC1*, while *AaFLC2* contributed the remaining 10% (S1C Fig); *AaFLC3* expression was undetectable in any population, so we did not study it further. From our genomic sequence data mapped to an updated BAC sequence of the *FLC* region (see Methods), we found no polymorphisms in *AaFLC2* in BGS relative to late flowering mountain populations, but one allele of *AaFLC1* segregating at about 20% in BGS has five non-synonymous derived polymorphisms relative to KA; two of these, located in exons 3 and 4, are unique to BGS. These amino acid changes lie within the functionally important K-box domain (positions 279 and 377 in CDS). We confirmed that these *AaFLC1* SNPs lie on the same haplotype by sequencing cDNA clones from KA and BGS individuals (Table 1). The derived allele is relatively rate, while the more frequent allele in BGS (80%) is identical to that found in late-flowering KA, and thus the encoded protein is likely functionally identical to that in KA and we did not study it separately.

**Table 1:** Polymorphisms in FLC1 cDNA clones between KA and BGS.

Position (CDS)	Exon	*A*. *lyrata*	KA	BGS	REF aa	ALT aa	Protein domain
6	1	G	G/A	G	-	-	
132	1	T	T	C	-	-	MADS
279	3	C	C	C/G	His	Gln	K-box
377	4	C	C	T	Thr	Ile	K-box
464	6	A	C/A	C	Glu	Ala	K-box
493	7	G	G	G/A	Glu	Lys	K-box
549	7	A	A/G	A	-	-	
566	7	C	C	C/T	Pro	Leu	

Shaded in grey are polymorphisms unique to BGS.”REF aa” indicates the ancestral amino acid and “ALT aa” the derived one.

To test whether the rarer *FLC* variant in BGS is functional, we isolated a cDNA of the BGS *AaFLC1* allele with the amino acid changes (*AaFLC1*^*BGS-d*^), *AaFLC2*^*BGS*^, and *AaFLC1*^*KA*^ and *AaFLC2*^*KA*^ from late-flowering KA. We expressed these under a constitutive promoter (35S) in the early flowering *A*. *thaliana flc*-*3* mutant (Fig 3C). We phenotyped over 30 independent transgenic lines for each of the four constructs for their leaf number at bolting (LNB). We considered only plants for which we could confirm expression by quantitative RT-PCR. For *35S*::*AaFLC1*^*KA*^, *35S*::*AaFLC2*^*KA*^ and *35S*::*AaFLC2*^*BGS*^ transgenic lines, LNB was significantly higher than the *flc-3* mutant, with *35S*::*AaFLC1* having a stronger effect. However, lines carrying *35S*::*AaFLC1*^BGS^ did not show a significant delay of flowering relative to the mutant, while those carrying *35S*::*AaFLC2*^BGS^ did (Fig 3). We measured *FLC* expression by qRT-PCR in each of the transgenic lines and for *AaFLC1*^*KA*^, *AaFLC2*^*KA*^, and *AaFLC2*^*BGS*^ and found a good correlation between *AaFLC* expression level and flowering time, suggesting these genes encode active versions of *FLC* (R^2^ > 0.4, p<0.001; S2 Fig) and that both *AaFLC1* and *AaFLC2* can function as floral repressors. For *AaFLC1*^*BGS*^, however, there was no correlation between transgene expression and flowering time (R^2^ = 0.02, p>0.5), suggesting that the amino acid changes render this rarer variant of *AaFLC1* non-functional, at least in terms of floral repression. This suggests that *FLC1* may be decaying in BGS. However, since this allele is found at only 20% frequency in the BGS population, it may contribute, but is not sufficient to explain that almost all BGS plants are early flowering; autotetraploid *A*. *arenosa* populations are in Hardy-Weinberg equilibrium [40], so at this allele frequency only 0.2% of plants would be homozygous for the derived allele (0.2^4^), while 41% of plants would be predicted to be homozygous for the active (late) ancestral allele (0.8^4^).

### Accelerated flowering induced by a derived *CO* allele

Since the QTL region in BGS x KA also contains *CO*, we asked whether *CO* alleles segregating in the BGS x KA F_2_s could contribute to early flowering in the BGS population. We sequenced the *CO* locus from early and late F_2_ individuals and identified two alleles differing by 14 SNPs in their coding sequence: one allele (hereafter *CO-A*) closely matches the ancestral state (based on comparison to related species *A*. *thaliana* and *A*. *lyrata* from which it differs by only two SNPs), while the other (*CO-B*) carried 12 independent derived polymorphisms relative to the *A*. *lyrata* allele. Out of the 14 sites that distinguish *CO-A* and *CO-B*, 9 are non-synonymous. Of these, *CO-A* has two and *CO-B* seven derived amino acid changes relative to *A*. *lyrata*.

We also identified an associated 7bp copy-number variant (CNV) in the promoter of *CO*: the *CO-A* allele carried five copies of the repeated GTGTAAA motif while the *CO-B* allele has only three. This CNV has previously been documented in *A*. *thaliana* populations and was shown to influence *CO* expression [41]. The expression difference in *A*. *thaliana* was proposed to result from different degrees of binding of CDF1, a day-time repressor of *CO* whose binding site (AAAG) is contained within the repeated motif [37,38]. Differences in expression between 4-repeat and 3-repeat promoters in *A*. *thaliana* were associated with differences in flowering time, with the 4-repeat promoter leading to later flowering.

In order to test if the two *CO* alleles segregating in BGS x KA have different phenotypic effects, we transformed the *A*. *arenosa CO-A* and *CO-B* alleles into late-flowering *A*. *thaliana co-9* null mutant lines (SAIL_24_H04). The transgenes contained the whole coding region including introns and 1.3kb upstream sequence. We grew >50 independent transgenic lines of each transgene alongside the *co-9* mutant in LD conditions. We phenotyped all plants for leaf number at bolting (LNB). Despite some variation in the flowering time of the transgenic lines (in particular CO-A: IQR = 12.3), both sets of transgenic lines flowered significantly earlier than the *co-9* mutant (p<1e-26), showing that both transgenes are functional. *CO-B* transgenic plants flowered earliest with a median LNB of 15.5 (IQR = 9.0, N = 56), and were significantly earlier flowering (p<1e-5) than *CO-A* plants which had a median LNB of 25 (IQR = 12.3, N = 60) (Fig 3D). That *CO-B* lines were significantly earlier than the *CO-A* lines, demonstrates there is a functional difference between the ancestral (*CO-A*) and derived (*CO-B*) alleles.

As we note above, *CO* is expressed more highly in BGS than in mountain plants, consistent with *CO-B* possibly playing a role in circumventing the repressive effect of high expression of *FLC* in BGS. Thus we asked whether this expression difference is a property of the two *CO* alleles themselves. For this we tested expression in our transgenic lines. Using qRT-PCR, we tested if the *CO-A* and *CO-B* transgenes in *A*. *thaliana* (which have 5-repeat vs 3-repeat of the putative CDF1 binding site in the promoters) showed a difference in expression. We measured *CO* expression in transgenic lines at mid-day (ZT8) and 1h before dark (ZT15). These 2 time-points were chosen as they represent the expected minimum (due to CDF1 repression) and maximum (due to GI activation) of *CO* expression respectively [41]. At mid-day (ZT8), *CO* expression is significantly lower in the *CO-A* transgenic lines than in the *CO-B* lines (p<1E-3) (Fig 3E), which is consistent with increased binding and repression by CDF1 of *CO-A*. A similar trend was observed at ZT15, though it was not significant, likely due to increased variation within lines (S6A Fig). These results suggest that the expression differences we observed in *A*. *arenosa* are caused, at least in part, by sequences that are contained in our transgenes. Good candidates are the CDF1 binding site repeats. The variation in expression among CO-A transgenic lines (log ratio of ZT8 over ZT15 expression) was significantly negatively correlated with variation in LNB (S6B–S6D Fig). A stronger but still relatively minor correlation (R^2^ = 0.1632, N = 51) could be observed between LNB and ZT8/ZT15 log ratio across both transgenes (S6C and S6D Fig), suggesting that expression differences of both alleles can affect flowering time.

### BGS: A mountain genotype with introgression of lowland railway alleles

**Fig 4:**
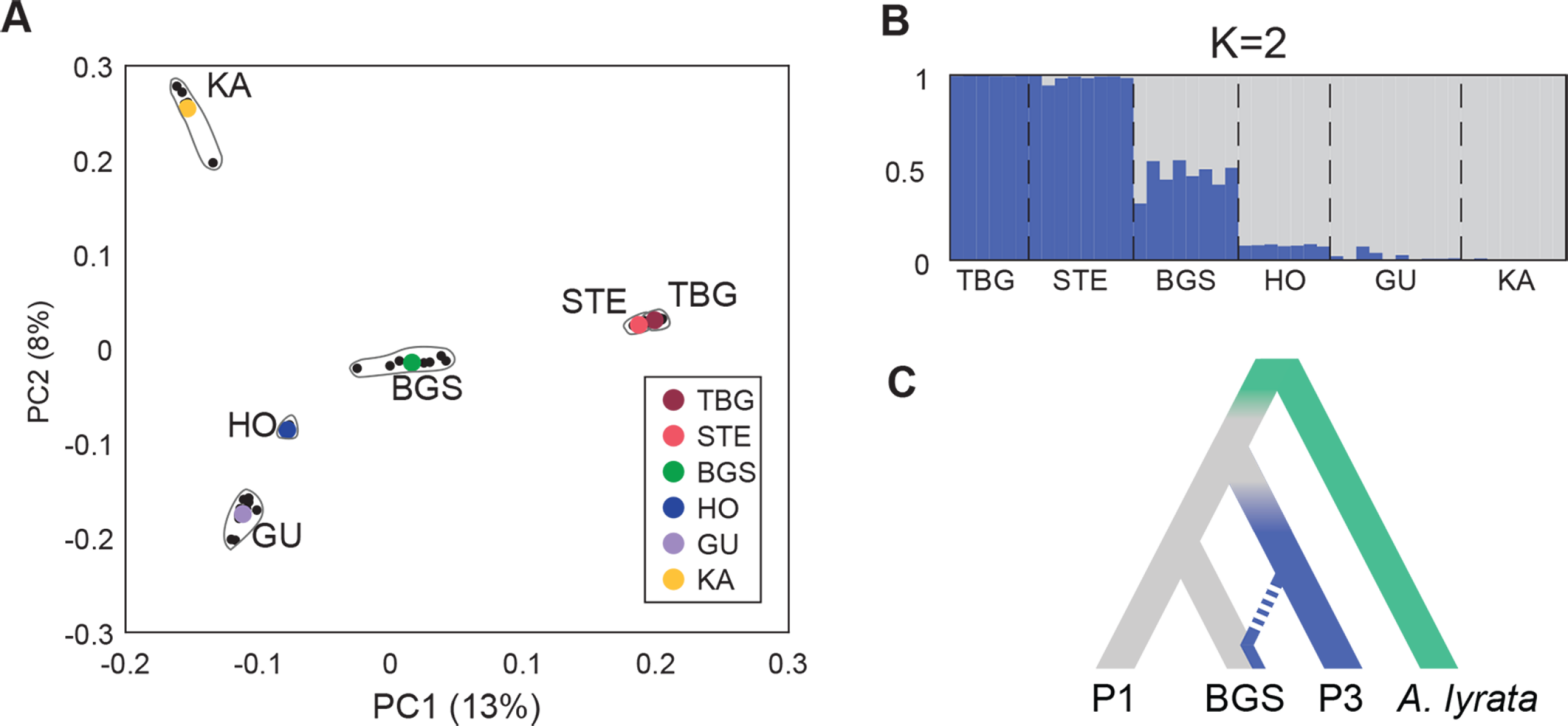
BGS shares genomic variation with lowland railway and mountain backgrounds. (A) First two principal components (PC1 and 2 with percentage of variance explained) of Principal Component Analysis (PCA) of the genomes from 47 re-sequenced individuals of three railway (TBG, STE, BGS) and three mountain (HO, GU, KA) populations. (B) Genomic clustering of individuals using STRUCTURE with K = 2. Each individual is represented by a single vertical line broken into K = 2 segment with length of each colored bar proportional to the posterior probability of belonging to each cluster. (C) Population history model used for ABBA-BABA evaluation of introgression fraction within BGS, with background and donor populations P1 and P3 and *A*. *lyrata* used as outgroup.

Having established that *CO* and to a lesser extent *FLC* play a role in the early flowering of BGS, we next sought to explore the genome wide patterns of selection and introgression in the BGS population. We already knew that although BGS is primarily a mountain genotype, it also has extensive shared polymorphism with the widespread lowland railway type (which is not true of other mountain populations [21]). To analyze the extent and patterns of this shared diversity and its potential role in adaptation, we complemented previously-generated whole-genome sequences [22,40,42] with additional individuals to reach a total of 47 individuals from BGS, two lowland railway populations (TBG and STE), and three mountain populations (HO, GU, and KA). PCA of genome sequence data recapitulated the pattern observed with the transcriptome: despite their geographic separation, lowland railway populations TBG and STE grouped tightly together and were clearly separable from mountain populations. BGS was again intermediate but closer to the mountains (Fig 4A). To further analyze population structure of our samples, we used STRUCTURE [43] on 627,016 SNPs. The ΔK ad-hoc statistics [44] support that these populations form 2 major groups comprising a railway clade including TBG and STE, and a mountain clade of HO, GU, and KA, while the 8 BGS individuals clearly showed a hybrid genomic constitution (Fig 4B).

We quantified the genome-wide fraction of introgression with the modified *f*-statistic ${\hat{f}}_{hom}$[45]. Using *A*. *lyrata* as a reference, we used either mountain or lowland railway populations as the donor population (P3) or the background population (P1) in the ABBA-BABA configuration (respectively columns and rows of Table 2, represented schematically in Fig 4C). We excluded GU because it has experienced gene flow from *A*. *lyrata* [42], which would bias estimates of introgression. When we used the mountain populations HO or KA as donors (P3), the estimates of introgression into BGS were ~20% higher than when we used these as background (P1), consistent with BGS being overall more mountain-like. If we thus assume BGS has a mountain origin, the fraction of introgression from the lowland railways was estimated between 8.7% and 11.9% (Table 2).

**Table 2:** Fraction of introgression ${\hat{f}}_{hom}$ with jackknife standard deviation.

		*donor (P3)*			
	$f_{hom}$	TBG	STE	HO	KA
*background (P1)*	TBG			30.40% (^+^/_-_ 0.5%)	26.26% (^+^/_-_ 0.41%)
	STE			33.03% (^+^/_-_ 0.48%)	28.12% (^+^/_-_ 0.37%)
	HO	9.75% (^+^/_-_ 0.4%)	11.91% (^+^/_-_ 0.63%)		
	KA	8.67% (^+^/_-_ 0.33%)	10.53% (^+^/_-_ 0.51%)		

We then used another metric for the fraction of introgression (${\hat{f}}_{d}$) to identify introgressed loci on a finer scale. ${\hat{f}}_{d}$ performs better than Patterson’s D and ${\hat{f}}_{hom}$ for identifying introgression on a small window basis [45]. For each mountain-railway (P1-P3) couple we calculated per gene estimates of ${\hat{f}}_{d}$ for any genes with more than 25 informative SNPs. We then scanned for genes with a high fraction of introgression (${{\hat{f}}_{d}>3*\hat{f}}_{hom}$) and kept only loci presenting high ${\hat{f}}_{d}$ in all 4 comparisons. This way we identified 1180 candidate introgressed loci from lowland railways into BGS (S4A–S4C Fig). We next asked if genomic regions introgressed from lowland railways into BGS had more railway-like expression profiles. Among the 1180 genes putatively introgressed from other lowland railway populations into BGS, only 74 (6%) were differentially expressed between lowland railway and mountain populations (defined excluding BGS). Of these, 6 genes (8%) have railway-like expression in BGS and 9 genes (7%) had mountain-like expression in BGS (S3 Table), showing that both cis and trans effects occur, and that overall there is no clear trend whether introgressed loci reflect the expression levels characteristic of the donor or the recipient.

### Differentiation of *CO* haplotypes

**Fig 5:**
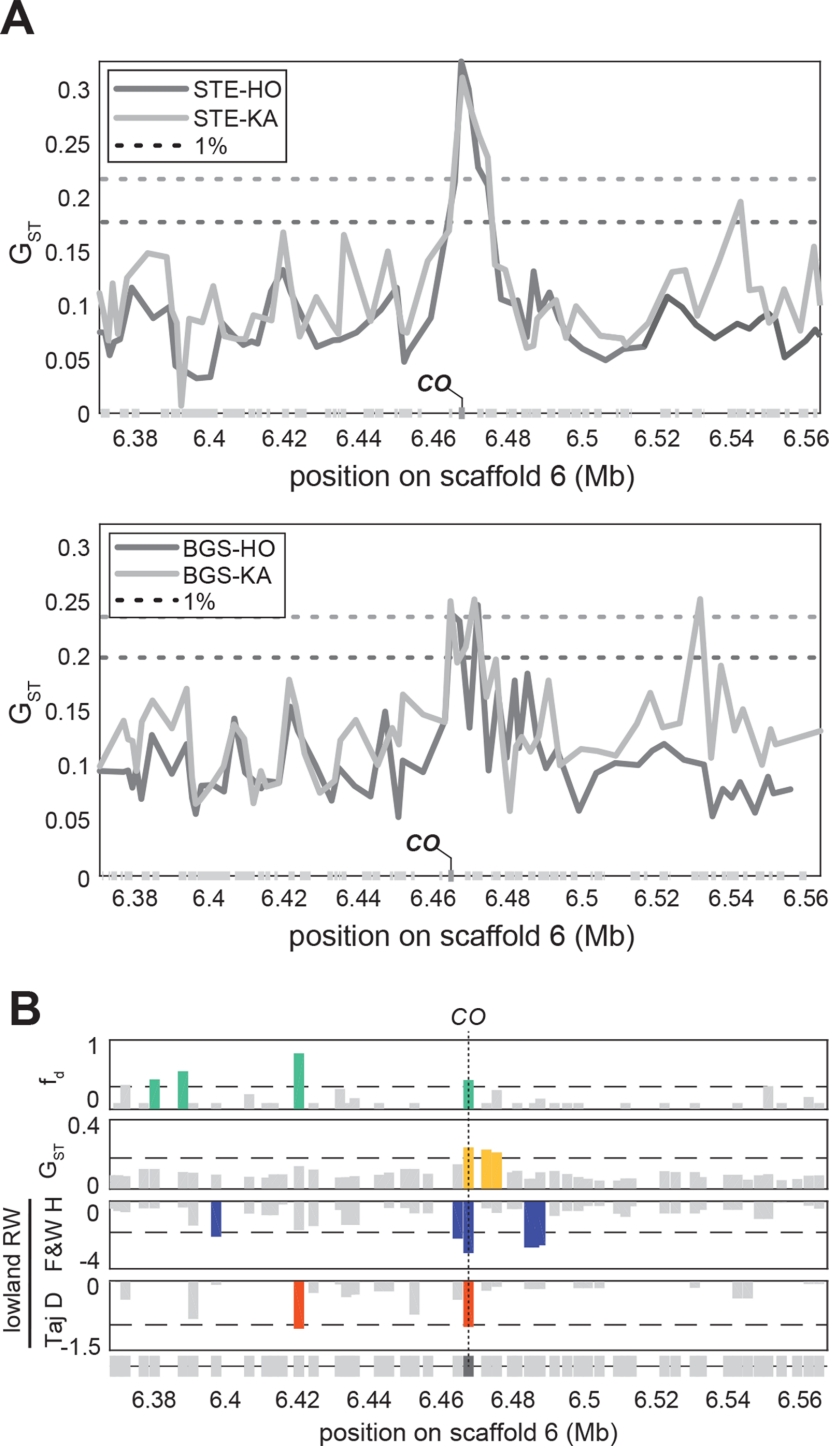
Railway-specific selection on a highly-differentiated railway haplotype of *CONSTANS*. (A) Marks of differentiation between one lowland railway population STE (upper panel) or BGS (lower panel) and two mountain populations (HO and KA) evaluated with G_ST_ across *CO* region. Dotted lines are respective genome-wide 1% threshold levels. (B) Gene-wise marks of introgression (${\hat{f}}_{d}$), railway-mountain differentiation (G_ST_), and railway-specific positive selection (Fay & Wu’s H, and Tajima’s D) across *CO* region. For each gene, only the least extreme values are represented. Dotted lines are $3*{\hat{f}}_{d}(KA,BGS,TBG)$ (upper panel) or most extreme genome-wide 5% threshold levels.

The difference in *CO* alleles found in BGS prompted us to examine its degree of differentiation among populations. First, we scanned the whole genome for regions with high genetic differentiation between lowland railway and mountain groups, excluding BGS. We identified 25 SNPs windows genome-wide within the top 5% for G_ST_, which is F_ST_ generalized to multi-allelic sites [46]; Figs 5A and S4A and S4D).

We then considered only windows that were outliers for Fay & Wu’s H, a statistic sensitive to excess high-frequency variants compared to neutral expectations [47], in only the lowland railway populations (TBG and STE), but not in the mountain populations. BGS was not included in this analysis since this population has had extensive introgression, which can bias Fay and Wu’s H [47]. By these criteria, 24 genes had marks suggesting railway-specific selection (S3 Table). Three of these genes were also outliers for Tajima’s D, which is sensitive to scarcity of low-frequency variants, a complementary mark that can indicate positive selection [48]. Even though the interpretation of both Fay & Wu’s H and Tajima’s D is complicated in populations with introgression, which may well be the case for lowland railway populations, the fact that *CO* fulfilled all three criteria (Figs 5B and S4A and S4E) suggests that *CO* may have been under selection also in the lowland railway populations.

**Fig 6:**
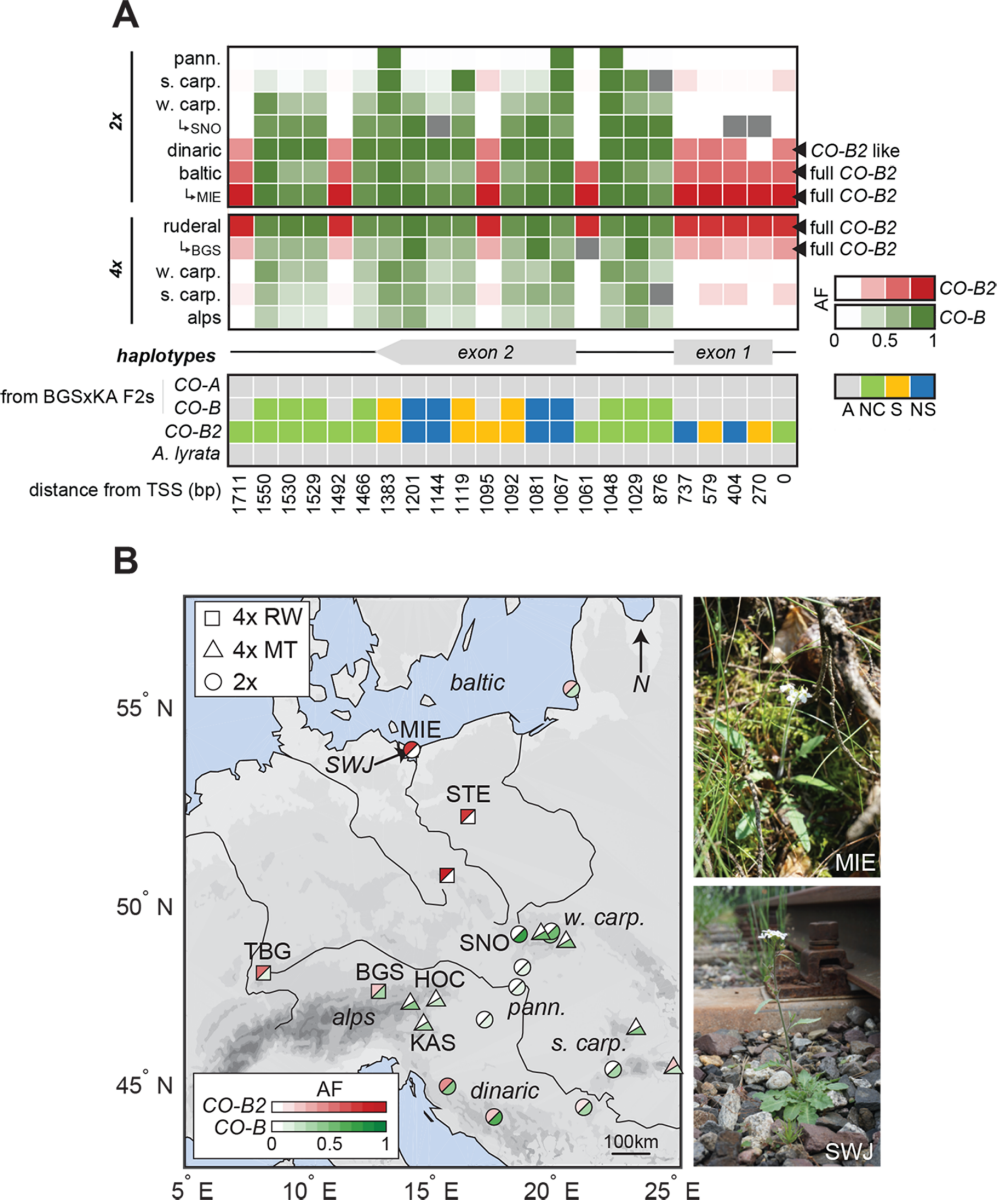
Origin of railway *CO-B2* in association with high-order CNVs found in diploids. (A) Upper panel: Average allele-frequency of 23 high-frequency railway *CO* variants in 5 diploid and 4 tetraploid clades. Population frequencies are detailed for SNO, MIE, and BGS which were differing significantly from their respective clades. High allele frequencies of the 9 signature railway polymorphisms (*CO-B2* allele) are in red and of the 14 *CO-B* polymorphisms in green. Lower panel: Comparison of the 3 *CO* haplotypes (*CO-A*, *CO-B*, *CO-B2*) along the 23 high-frequency railway *CO* variants color-coded by impact on the coding sequence (non-synonymous in blue, synonymous in yellow, and non-coding in green) compared to *A*. *lyrata* (grey). (B) Map of 11 tetraploid and 12 diploid populations from Monnahan et al. color-coded by their average frequency of *CO-B2* signature polymorphisms in red and *CO-B* polymorphisms in green. Pictures of diploid MIE and the adjacent tetraploid railway population SWJ during July 2017 field collections from likely interploidy introgression region on the Baltic.

*CO* alleles that predominate in mountain and lowland railway populations are clearly distinct (Fig 5A). Within 200bp of the coding sequence and across the intron, we found 27 derived polymorphisms (relative to the reference *A*. *lyrata* genome [24]) with frequency differences higher than 30% between lowland railway and mountain populations. Of the 27 polymorphisms that distinguish these alleles, 23 are in the derived state in the lowland railway lineage. We found, however, that this lowland allele of *CO* is to some extent distinct from the *CO-B* allele that we previously showed contributed to early flowering in BGS x KA crosses. Fourteen of the 23 derived polymorphisms that characterize the lowland railway allele are shared with the *CO-B* allele (Fig 6A), thus we conclude that this allele arose from the *CO-B* allele through the acquisition of nine additional derived mutations, and is not independently derived from *CO-A*. To reflect this likely shared ancestry, we named the lowland railway allele *CO-B2*. In these populations, the nine SNPs characteristic of *CO-B2* segregate at frequencies averaging 79% (sd = 0.03) in both STE and TBG and around 30% in BGS. SNPs characteristic of *CO-B* are present but rare in mountain populations, while those characterizing *CO-B2* are absent from the mountain populations sampled. One of the 23 high-frequency railway polymorphisms is at the transcription start site (TSS) and six others are predicted to cause non-synonymous amino-acid substitutions, including two of the nine SNPs unique to lowland railways.

### Origins of *CO-B* and *CO-B2*

The relationship of *CO-B* and *CO-B2* prompted us to explore the biogeographic pattern of these alleles to better understand their evolutionary histories. To do this, we extracted and genotyped the 10kb region surrounding *CO* from whole-genome sequences (Monnahan et al. submitted) of a broader sample of tetraploid and diploid clades. This dataset includes 172 individuals from 11 tetraploid and 12 diploid populations (respectively 87 and 85 individuals) from across the *A*. *arenosa* range (Fig 6A and 6B and S1 Table). This analysis indicated that among tetraploids, *CO-B* is found as a rare variant in some mountain populations, but reaches a relatively high frequency in BGS of around 30% (Fig 6B). *CO-B2* is at very high frequency in all lowland railway populations (STE, KOW, TBG), completely absent from mountain populations in the Alps and Carpathians, but found in BGS at frequencies around 30%. Only in BGS do both the *CO-B* and *CO-B2* alleles appear at intermediate frequency (Fig 6A and 6B). At 30% frequency, 24% of individuals would be lacking either *CO* allele, but only 2.6% would be homozygotes for *CO-A*. Thus if both *CO-B* and *CO-B2* have dominant or semi-dominant effects on flowering, the presence of these alleles could explain much of the early flowering of the BGS population.

Among diploids, however, the picture is more complex, with several intermediate alleles present. These are informative. Diploids from the Pannonian basin carry three of the 14 derived polymorphisms characteristic of *CO-B*, while diploids from the southern Carpathians gain two more high-frequency *CO-B* polymorphisms. West Carpathian diploids, the closest relatives of the tetraploids [21], carry all but one of the *CO-B* polymorphisms at moderate to high frequency (Fig 6A). Dinaric populations carry *CO-B*, as well as all but two of the polymorphisms characteristic of *CO-B2*. Diploid populations along the Baltic, however, carry all of the polymorphisms characteristic of *CO-B2* and one population, MIE, was nearly fixed for *CO-B2* (Fig 6A and 6B).

Since tetraploids have been shown to have originated from West Carpathian diploids [21], where *CO-B2* is virtually absent, railway tetraploids most likely obtained their derived *CO-B2* haplotype through secondary contact and interploidy introgression in the Baltic region. Such interploidy gene flow is known to occur in *A*. *arenosa* among proximal populations [21,49]. We cannot entirely rule out that *CO-B2* originated in the railway tetraploids and introgressed into Baltic diploids where it subsequently came under selection, but introgression from tetraploids to diploids is very rare in *A*. *arenosa* compared to the reverse [21], and the existence in diploids of alleles intermediate between *CO-B* and *CO-B2* (Fig 6A) further supports the notion that *CO-B2* evolved through the progressive accumulation of derived polymorphisms on a *CO-B* background in diploids. The MIE population, which has the highest frequencies of the *CO-B2* signature polymorphisms (averaging 0.88), is found on the Baltic coast at the border between Poland and Germany. We visited Northern Poland to investigate whether tetraploid railway populations were present in the area surrounding MIE and indeed found large populations of tetraploid *A*. *arenosa* on railbeds within 10 km of the diploid MIE population (SWJ in Fig 6B). This close proximity supports the hypothesis that there could be a secondary-contact zone on the shores of the Baltic from which the diploid *CO-B2* haplotype could have entered the railway tetraploid lineage via interploidy introgression where it subsequently came under selection.

## Discussion

We focused here on studying apparently independent colonisations of a ruderal habitat–railway beds–in the otherwise non-ruderal plant, *Arabidopsis arenosa*. Using genome and transcriptome data, we investigated the extent and consequences of gene flow among ruderal populations. We also identified genes involved in the evolution of a key ruderal trait, rapid cycling, and studied the history of the causal alleles. In contrast to mountain populations, most lowland railway populations within autotetraploid *A*. *arenosa* form a single genetic lineage that spread over hundreds of kilometers across central Europe, consistent with the idea that railways and roadsides provide “corridor” habitats that can facilitate rapid dispersal of adapted colonists (e.g. [1–5, 7, 15, 16]). We found that one population we sampled from a mountain railway site in Berchtesgaden, Germany (BGS) may have colonized railways independently, but sustained substantial gene influx from previously existing lowland railway populations.

### Genetic basis of early flowering in *A*. *arenosa*

Autotetraploid *Arabidopsis arenosa*, like its diploid progenitor, is generally perennial and found on rock outcrops and scree in mountainous and hilly regions throughout much of Europe. Almost all are late flowering when grown in laboratory conditions [22]. However, one unique genetic lineage colonized railways throughout central and northern Europe [21], and, befitting ruderal plants, members of this lineage are rapid cycling and perpetually flowering annuals [22]. Here we explored the genetic mechanisms underlying the evolution of rapid cycling in *A*. *arenosa*, focusing in particular on a population in the Northern Alps that is clearly a hybrid of mountain and widespread railway types (BGS). We initially hypothesized that this population had adapted to railways by appropriating genes from the lowland railway lineage, but while introgression has clearly contributed potentially adaptive alleles, there is also evidence of independent adaptation having occurred within this population.

We found both here and previously that the widespread lowland railway *A*. *arenosa* plants have lost expression of the floral repressor *FLC* (this study and Baduel et al. [22]), which likely explains their early flowering. In our mapping experiments, markers close to *FLC* were strongly associated with additive effects on flowering time, especially when one parent was one of the widespread railway lineage (TBG), but also when using the mountain railway parent (BGS). This parallels findings in *A*. *thaliana*, where loss of *FLC* is seen in early flowering accessions [26–33], as well as *A*. *alpina*, where loss of the *FLC* homolog *PEP1* is associated with a switch from episodic flowering and a requirement for vernalization, to rapid cycling and perpetual flowering [50,51].

Our initial hypothesis was that BGS, which is as early flowering as the lowland railway populations with which it hybridized, acquired an early flowering *FLC* allele through gene flow from the lowland railway populations. However, this turned out not to be the case; unlike the lowland railway populations, the equally early BGS plants retain high levels of *FLC* expression, suggesting the mechanism of early flowering in BGS and the lowland railway populations is at least to some extent distinct. High expression of *FLC* is usually associated with late flowering [26], as well as low expression of a key reproductive transition promoting gene, *SOC1* [52]. We found, however, that despite its high *FLC* expression, BGS also has high *SOC1* expression, consistent with its early flowering. But how could BGS express high levels of *SOC1* and flower early while still having high expression of *FLC*? We considered two scenarios: (1) BGS could be expressing a non-functional allele of *FLC*, or (2) BGS could express an active allele, but somehow circumvents *FLC’s* repressive activity. To test the first idea we functionally tested the different *FLC* alleles found in BGS, we found that one expressed *AaFLC1* allele in BGS is inactive at least with respect to delaying flowering, suggesting FLC is decaying in this population, giving support to scenario 1 above. But while this allele can affect flowering time, it is found in BGS at only 20% frequency. The more common allele is identical to the active alleles found in other mountain plants. This suggests *FLC* is likely a relatively minor player in the early flowering of BGS plants overall, and not the whole story of early flowering in BGS (even if this derived allele were fully dominant, 40% of plants would still be homozygous for the ancestral late allele, which does not fit with observation of flowering time).

Our mapping, gene expression data, scans for differentiation and selection, as well as functional follow-up, support the hypothesis that an additional gene responsible for the early flowering of BGS may be *CO*. *CO* has higher levels of expression in BGS and maps well within the high confidence QTL region in the BGS x KA cross. *CO* is known from *A*. *thaliana* to be a direct regulator of the flowering promoting gene *SOC1*, which it targets antagonistically with *FLC* [53]. Importantly, in *A*. *thaliana* it has been shown that high CO activity can circumvent high FLC activity to activate *SOC1* and promote flowering [38]. We found that BGS carries two derived *CO* alleles. One of these (*CO-B*) is found as a rare variant in other mountain populations where it is present but very rare. This allele segregates in our BGS x KA F_2_s, and we showed with transgenics in *A*. *thaliana* that it confers higher *CO* expression and earlier flowering relative to the ancestral *CO-A* allele. *CO-B* is at higher frequency in BGS than any other mountain populations. A second derived *CO* allele (*CO-B2*), which is related to the *CO-B* allele, is also found at moderate frequency in BGS (30%) while it is absent from other mountain populations. Our biogeography data suggest that the *CO-B2* allele likely arose in diploid populations and entered the railway gene-pool over 700 km away along the Baltic Sea. After arriving via interploidy gene flow, the *CO-B2* allele seems to have swept through the lowland railway populations, almost completely replacing *CO-B*, and ultimately found its way to BGS.

Numerous other genes also introgressed into BGS from lowland railways. Some alleles that arrived by gene flow may have subsequently came under selection in BGS, but one of the most strongly differentiated genes following introgression is the *CO-B2* allele of *CO*, suggesting it came under selection post-hybridization. The *CO-B* and *CO-B2* alleles differ from the ancestral allele (*CO-A*) that predominates in mountain populations by 14 and 23 linked polymorphisms, respectively, with *CO-B2* containing all 14 of the *CO-B* polymorphisms. Several of the *CO* polymorphisms cause amino acid changes, and a subset of these are unique to the railway *CO-B2* haplotype. In BGS, the finding that *CO-B2* may be under selection even though the early flowering *CO-B* allele (which seems to have been selected from a rare variant from the mountain gene pool) is also present, suggests *CO-B2* may either be more effective or has some other benefit. Understanding whether *CO-B2* is equivalent to CO-B, is in the process of outcompeting *CO-B*, or confers other benefits requires more investigation. The involvement of CO leads us to speculate that the apparent loss of function allele of *FLC* that segregates in BGS is likely a decay of function allele of a now unnecessary gene, rather than the primary cause of early flowering in BGS. Overall, we hypothesize that *CO* is an important factor for early flowering in BGS since most plants carry either *CO-B* or *CO-B2 –*at the frequencies at which these alleles are found, only 2.5% of plants would carry neither of them. In our mapping data, it was clear that the *CO* region has quantitative effects on flowering. What the function is of the *CO-B2* allele, or what other differentiated genes might be functionally important for ruderal adaptation, remains to be explored.

### A mixed history of *CONSTANS* alleles in *A*. *arenosa*

The *CO-B2* allele also shows evidence of having been under selection in the lowland railways. This raises the possibility that it may have played a role in flowering regulation in these populations as well, with the loss of *FLC* following later (as now seems to be happening in BGS). Alternatively, *CO-B2* may confer some other benefit even in the context of an already early genotype. Like *FLC*, *CO* has also been implicated in a range of species in natural variation for flowering time, including *A*. *thaliana*, *Brassica nigra*, and rice [52–54,42], this additional example bolsters the idea that like *FLC*, *CO* can be an evolutionary hotspot for flowering time.

Our results indicate a mixed story for the evolution of early flowering in BGS and more broadly of the *CONSTANS* gene in *A*. *arenosa*. In BGS, one of the two major *CO* alleles seems to have been selected from standing variation already present in mountain populations (*CO-B*), while the other (*CO-B2*) arrived via gene flow from a widely distributed ruderal *A*. *arenosa*. The railway populations of *A*. *arenosa* are all tetraploids, as are the mountain relatives of BGS where the *CO-B* allele is found, but both *CO-B* and the *CO-B2* allele derived from it, seem to have originated in diploids. An allele almost identical to *CO-B* (missing only one of the 14 derived mutations that mountain tetraploids have relative to *CO-A*) is present in the Western Carpathian diploids, the closest relatives of all the tetraploids [21] and was thus likely carried into the mountain gene pool during the polyploidy event or subsequent hybridization with ancestral diploids. Alleles intermediate between CO-B and CO-B2 are found in several diploids, but the *CO-B2* allele found in the widespread lowland railways is present in diploids along the Baltic coast. We believe the presence of intermediate alleles in diploids hints that the *CO-B2* allele arose in diploids, and entered the lowland railway tetraploids via interploidy gene flow, which we know from previous work can occur in *A*. *arenosa*, primarily from diploids to tetraploids [21].

### Gene flow, *de novo* mutation and standing variation in ruderal colonization by *A*. *arenosa*

BGS clearly had considerable gene influx via hybridization, and at least some introgressed loci (including CO) increased in frequency, suggesting they came under selection. These findings add to a growing body of evidence that adaptive introgression may be an important factor in rapid adaptation to challenging environments [43,55–61]. The loss of *FLC* function on an expressed haplotype unique to BGS (within our sampling) seems to be a novel mutation. Thus the colonization of this ruderal habitat by the BGS plants seems to have utilized multiple allele sources including introgression via hybridization (*CO-B2*), selection from standing variation (*CO-B*), and *de novo* mutation (*FLC1*). The spread of *CO-B2* after introgression additionally highlights how “corridor ruderals” (weedy variants found on linearly extended human-generated habitats such as roadsides or railways) can alter the genetic architecture and adaptive potential of plant species by facilitating gene flow among otherwise isolated populations. In this case *CO-B2* alleles originating in diploid populations seem to have found their way via the lowland railways to a population over 700km away in the Alps.

### Railway *A*. *arenosa* as a corridor ruderal that can facilitate gene flow

The rail networks in Germany and Poland became widely connected in the mid to late 1800’s (S5 Fig), but the widespread “lowland railway lineage” seems to have diverged from other *A*. *arenosa* earlier than that [21], suggesting it inhabited a similar habitat elsewhere (e.g. mountain scree slopes, river cobbles, or perhaps ancient agricultural settings) that allowed it to rapidly colonize railways as the networks were built. Subsequent spread of *A*. *arenosa* along railways then allowed contact between genotypes that were previously geographically isolated (as most modern mountain *A*. *arenosa* genotypes are [42,62]) and thus the lowland railway lineage acquired (with inadvertent human assistance) the potential to act as a conduit of gene flow. Colonization of the Berchtesgaden railway where the BGS population is found was likely quite recent compared to the colonization of other railways. The railway to Berchtesgaden was built in 1888, but completely rebuilt in 1940 to accommodate sudden heavy traffic to Hitler’s infamous Eagle’s Nest, built above Berchtesgaden in 1937.

The BGS population is primarily mountain-like, both in terms of genome sequence and gene expression, but has sustained substantial gene flow from lowland railway plants. We note that although we treat BGS as a mountain colonist of railways, we cannot rule out that it might not have gone the other way–namely that BGS might have been first colonized by a railway type that was then genetically “swamped” by gene flow from adjacent mountain populations. In either case, the observation that numerous genes in BGS have become, or remain, distinctly railway-like in this population, suggests that in this “mountain railway” population, selection acted to favor some alleles of lowland railway origin in an otherwise mountain genome. This highlights the potential for introgression of alleles from widespread lowland railway ruderals into neighboring non-ruderal populations that may play a role in secondary colonization, and also that “corridor ruderals,” by allowing the spread of alleles across large geographical distances, can affect the adaptive process in local populations they contact.

These findings support the idea that some (but not all) adaptive alleles in BGS and in lowland railway tetraploids arrived by gene flow and is consistent with a growing number of examples of “adaptive introgression” having played a role in local adaptation [55–61]. On the other hand, in cases like BGS, the new colonist brings novel alleles from its original mountain home to the lowland railways, and in follow-up work it will be interesting to ask whether adaptive introgression is a two-way street.

## Materials and methods

### Plant materials and growth conditions

All *A*. *arenosa* materials used in this study are autotetraploids previously described by Baduel et al. (S1 Table). We grew sibling arrays from seeds of single individuals in nature as previously described [42] in Conviron MTPC-144 chambers with 8 hours dark at 16°C, 4 hours light (Cool-white fluorescent bulbs) at 18°C, 8 hours light at 20°C, 4 hours light at 18°C. For all plants we recorded germination date by root emergence on agar ½ X Murashige-Skoog plates. We also grew *A*. *thaliana* plants in Conviron MTPC-144 chambers, but with 16 hours light (Cool-white fluorescent bulbs) and 8 hours dark at constant 22°C.

### Genetic mapping

We generated F_2_ populations from both TBG x SWA and BGS x KA crosses by intercrossing F_1_ siblings and phenotyped all plants for flowering time using time to bolting (defined as the time when the inflorescence reached 1 cm tall). For plants that had not flowered by experiment end (80 days for TBG x SWA and 140 days for BGS x KA) we assigned these end dates as cutoff values. We then genotyped 130 individuals at each end of the phenotypic distribution (~15% of F_2_s) using RADseq. We prepared sequencing libraries using a modified double-digest RAD-seq protocol as previously described [21]. We sequenced libraries on an Illumina HiSeq 2000 with 50 bp paired end reads, to 16x coverage. We only considered SNPs present in a minimum of 40% of individuals, and obtained LOD scores and p-values for each SNP from a simple marker linear regression analysis. For each regression we compared additive, recessive, and dominant models and used the model providing the highest LOD. Within the high-LOD markers (LOD>30) of scaffold 6, we then ran a stepwise multiple linear model (MLM) regression and discarded markers not significantly improving the sum of squared errors (F-statistics).

### RNA isolation, sequencing and analysis

We extracted RNA from leaves collected 9h after dawn (Zeitgeber time) from three-weeks-old (i.e. 30 days before the earliest flowering time) plants grown as described above with three biological replicates for each of seven populations (TBG, BGS, STE, KA, CA2, HO, SWA) using the RNeasy Plant Mini Kit (Qiagen). We synthesized single strand cDNA from 500ng of total RNA using VN-anchored poly-T [23] primers with MuLV Reverse Transcriptase (Enzymatics) according to the manufacturer’s recommendations. We made RNAseq libraries using the TruSeq RNA Sample Prep Kit v2 (Illimina) and sequenced libraries on an Illumina HiSeq 2000 with 50bp single-end reads. We sequenced between 9.8 and 18.8 million reads (avg 13.6 million) per individual. We aligned reads to the *A*. *lyrata* genome [24] using TopHat2 [63] and re-aligned unmapped reads using Stampy [64]. We acquired read counts for each of the 32,670 *A*. *lyrata* gene models [24] using HTseq-count [65]. We assessed quality of the count libraries by PCA and Euclidean distance analysis; biological replicates of each population are most similar to each other (Fig 1C). We normalized for sequencing depth using DEseq2 in R [66] and further analyses were performed in MATLAB (MathWorks). PCA was performed using the 500 genes with the highest variability, as recommended in the DESeq2 package [66].

The *FLC* locus in *A*. *arenosa* has three tandemly duplicated *FLC*-like genes [39,67] of which two, *AaFLC1* and *AaFLC2* are clearly homologous to *FLC* from *A*. *thaliana*. RNAseq reads from the *A*. *arenosa FLC* region do not all differentially map to the two *FLC* duplicates of *A*. *lyrata* so we also aligned transcriptome reads to the BAC sequence of the *FLC* region [67] updated to better discern read counts for each *FLC* as described in Baduel et al. [22].

Correlations between flowering time and gene expression were calculated between the average non-vernalized flowering time reported in Baduel et al. [22] and the average expression of each gene per population, after filtering for genes with normalized expression counts above 10 in at least one sample to avoid low expression artefacts. We excluded BGS to obtain the overall correlation coefficient for each gene. We obtained a list of 76 “flowering-correlated” genes (S2 Table) by retaining the top 1% most strongly correlated after filtering for genes with significantly different expression between mountain and lowland railway plants (at p < 0.05). We then asked whether the BGS datapoint falls outside the 95% confidence interval of the regression line and calculated how likely its position is given the noise in each trend (as we did for *FLC*, Fig 1D). For each gene we estimated how likely this BGS residual could be obtained from a distribution of residuals modeled as a normal distribution of mean 0 and sigma estimated as the standard-deviations observed with all other populations (two-tailed comparison).

### Differentiation analysis

To test for genetic differentiation, we used our previously published genomic short read sequences for *A*. *arenosa* [42,68] complemented with similarly processed genomes to reach 6 TBG, 8 STE, 8 BGS, 10 GU, 7 HO and 8 KA individuals for a total of 47 individuals over 6 populations. We aligned reads to the *A*. *lyrata* genome [24] using BWA [69] and re-aligned unmapped reads using Stampy [64]. We calculated G_ST_ [70], Fay and Wu’s H [47] and Tajima’s D [48] over 25 SNPs windows using customized scripts (available at: github.com/baduelp/public) after genotyping the alignments with GATK [71] only considering bi-allelic sites with a sequencing depth per individual of 4 or more (2.9 million SNPs).

For population structure analyses, we performed PCA on MATLAB (MathWorks, and script available at github.com/baduelp/public) and we used STRUCTURE [43] version 2.3.4 on 627 016 SNPs with a sequencing depth per individual of 8 or more (increased for computing memory purposes) with K values (number of groupings) ranging from 1 to 6.

We calculated both Patterson’s *D*-statistic and modified *f*-statistics (${\hat{f}}_{hom}$ and ${\hat{f}}_{d}$) were calculated as described by Martin et al. [45] (see S1 Text).

For graphic representation (Fig 5B), gene-wise estimates of G_ST_, Fay & Wu’s H, and Tajima’s D were obtained using the most extreme value of all windows overlapping a gene annotation. We then used the least extreme values of gene-wise G_ST_ calculated between the four railway-mountain couples (STE-HO, STE-KA, TBG-HO, and TBG-KA) as estimates of railway-mountain differentiation, the least extreme values of gene-wise Fay & Wu’s H and Tajima’s D in STE and TBG, to plot along the least extreme gene-wise ${\hat{f}}_{d}$ estimates obtained for each of the four railway-mountain couples.

### Cloning and transgenics

For *FLC* constructs, we synthesized single strand cDNA from 500ng of total RNA of BGS and KA and PCR-amplified both *AaFLC* with primers 5’-CCCTCTCGGAGACAGAAGCCATGG-3’ (forward) and 5’-AGGTGGCTAATTAAGCAGCGGGAGAGTCAC-3' (reverse). For *CO*, we PCR-amplified the locus including 1.3kb upstream from gDNA of KA x BGS F_2_s using primers 5’-GCATAGAGTGAAGGAAGCCACT-3’ (forward) and 5’- AGAAAGCACGCGGATGCATA-3’ (reverse). We then cloned the PCR products into pBluescript and sequenced using M13 primers (for FLC) and for CO, the primers 5’-GACTACTTGGCGGATTCGAGT-3’ (800bp upstream), 5’-GCAAGTGGCAAAACCTAAGC-3’ (273bp upstream), 5’-TGATGCTCAAGT-TCACTCTGC-3’ (1^st^ exon), 5’-ATCAACACCAGCAAAACTGCG-3’ (1^st^ exon), and 5’-AAGCAAGGTGAAATCTGTGT-3’ (259 bp downstream). We then cloned the transgenes into pGREEN [72] with the CMV 35S promoter (FLC only; 35S was removed for CO) and the Rbsc terminator.

We transformed confirmed *FLC* constructs into *A*. *thaliana flc-3* mutants in the Col-0 genetic background (kindly donated by R. Amasino) and *CO* constructs into Col-0 *co-9* mutants (SAIL_24_H04; kindly provided by P. Salomé) using *Agrobacterium tumefaciens*, strain GV3101 by floral dipping [73]. We selected first generation transformants (T1) on 1/2X MS plates with kanamycin (50 ug/ml) and transferred resistant seedlings to soil after one week and phenotyped by counting leaf number at bolting (LNB). We collected leaf-tissue from all T1 plants at 3-weeks post germination (for CO, two time points were collected, ZT15 at 3 weeks and ZT8 at 6 weeks) and quantified transgene expression by qRT-PCR on a Mx3005P machine (Stratagene) for *FLC* and for *CO* a CFX96 machine (BIO-RAD) using LightCycler 480 SYBR Green I Master (Roche). We used an annealing temperature of 55°C using Taq DNA polymerase (New-England BioLabs). We carried out reactions in triplicate, and normalized expression against expression of *ACTIN* using the 2^–ΔΔCT^ method, taking into account each primer’s efficiency as described in the BIO-RAD Real Time PCR Applications Guide. The standard deviation of each biological replicate was calculated using a first order propagation of error formula on the variance of the technical replicates. For *FLC* we used cDNA-specific primers 5’-CAGCTTCTCCTCCGGCGATAACCTGG-3’ and 5’-GGCTCTGGTTACGGAGAGGGCA-3’ (87% efficiency) and for *ACT* we used 5’-CGTACAACCGGTATTGTGCTGGAT-3’ and 5’-ACAATTTCCCGCTCTGCTGTTGTG-3’ (91% efficiency). For *CO* we used cDNA-specific primers 5’-TGTGTTCGTTATGGTTAAGGG-3’ and 5’-ATCAACACCAGCAAAACTGCG-3’ for *CO* (99.1% efficiency on *CO-A* and 84.6% on *CO-B*) while *ACT* had 112% efficiency in this experiment.

### Analysis of the *CO*-locus from whole-genome resequencing

We analyzed the *CO-*locus in a dataset of whole-genome sequences of 172 individuals from 11 tetraploid and 12 diploid populations (respectively 86 and 85 individuals) assembled by Monnahan et al. (submitted) in addition to the 47 genomes we already had. Population information is given in S1 Table. We aligned reads to the *A*. *lyrata* genome [24] using BWA [69] and genotyped with the GATK HaplotypeCaller and VariantFiltration using the filters:

‘QD<2.0||FS>60.0||MQ<40.0||HaplotypeScore>13.0||MappingQualityRankSumTest<-12.5||ReadPosRankSum<-8.0’. We obtained population allele frequencies after polarizing reference alleles against a panel of 23 *A*. *lyrata* genomes to avoid miscalling of variants considering only bi-allelic sites with a sequencing depth per individual of 4 or more in a minimum of 5 individuals. For the frequency analysis of the 23 derived railway polymorphisms (Fig 6A). We directly inferred allele frequency of missing sites (6.7 per population on average) from BAM read-counts calculated from reads with MQ>40 and DP>4. For non-missing sites, the correlation between BAM read-counts and genotyped frequencies was >92%.

We calculated the neighbor-joining tree of the *CO-*locus from 100 bootstraps of the alignment of the consensus *CO* region including 2kb upstream and 200bp downstream for all 172 individuals under a Tamura-Nei genetic distance model using the *A*. *lyrata* reference sequence as an outgroup.

### Accession numbers

RNAseq read data and RADseq mapping data have been deposited in the NCBI SRA database under accession number SRP148726 within the NCBI BioProject PRJNA472485. Custom scripts used are available at: github.com/baduelp/public.

## Supporting information

S1 Text Methods for distribution of percentages of variance explained (PVE) and modified f-statistics. (PDF)

S2 Text Methods for haplotype inference from RADseq genotypes. (PDF)

S1 Fig Differential expression between railway and mountain accessions. (A) Volcano plots of differential expression (q-value) against log expression ratios between railway and mountain accessions (excluding BGS) within whole transcriptome. 5% most differentially expressed (two-tailed log-ratio) are highlighted in green and within these, flowering-time genes (FT) are in red. (B) Volcano plots of differential expression (q-value) against log expression ratios between railway and mountain accessions (excluding BGS) within FT-peak region. 5% most differentially expressed (two-tailed log-ratio) are highlighted in green and within these, flowering-time genes (FT) are in red. (C) Paralogue-specific FLC expression: relative expression of AaFLC1 (light grey) and AaFLC2 (dark grey) across mountain populations and BGS.
(PDF)

S2 Fig Correlation between flowering time and transgene expression. (A, B) Correlations between flowering time, measured as leaves number at bolting (LNB), and relative FLC expression in transgenic T1 lines for AaFLC1 and AaFLC2 35S-driven cDNA transgenes of KA (A) and BGS (B). The regression line is represented in dotted line surrounded by the confidence intervals (shaded area). Black triangles mark the two late-flowering individuals obtained with BGS AaFLC1 transgenes. Lines where transgene expression was below 50% of ACT expression (<0.5) are hollowed out.
(PDF)

S3 Fig Phenotypic distribution of flowering time and PVE distribution across the FLC-CO region in TBG x SWA and BGS x KA F2s. (A, B) Distribution of flowering time (Days to Bolting) in phenotyped (grey) and sequenced (blue) in TBG x SWA (A) and BGS x KA (B) F2 individuals. (C) Distribution of percentages of variance explained (PVE) across the FLC-CO region in TBG x SWA and BGS x KA. PVE distributions are shown for each cross above the gene models for the region. Single marker model (SMM) percent variance explained (PVE) are plotted in grey on the primary (left) y-axis, while semi-partial correlation coefficients (SPC) from the multiple linear model are in blue against the secondary y-axis.
(PDF)

S4 Fig Marks of railway-specific selection and introgression. (A) Distribution of whole-genome (grey) and outliers values of mean fd across all 4 RW-MT pairs (green, outer ring), mean GST across all 4 RW-MT pairs (yellow, middle ring). (B,C) Distribution of introgression tract lengths for TBG-BGS-KA and STE-BGS-HO respectively. (D) Marks of differentiation between one railway population (TBG) and two mountain populations (HO and KA) evaluated with GST across CO region. Dotted lines are respective genome-wide 1% threshold levels. (E) Fay and Wu’s H on 200kb region surrounding CO, with genome-wide 5% threshold levels (dotted lines).
(PDF)

S5 Fig Rail networks in central Europe from 1849–1861. Map showing the rail network in Germany and surrounding areas from 1849. The railways are indicated as solid bold black lines. Lines added by 1861 are shown as dotted lines illustrating the rapid expansion of a widely connected transport network. Map image is public domain and obtained from Wikipedia: https://en.wikipedia.org/wiki/History_of_rail_transport_in_Germany.
(PDF)

S6 Fig CO expression in CO-A and CO-B transgenic lines. (A) Normalized CO expression at ZT15 of CO-A (orange) and CO-B (blue) T1s. (B-C) Correlations between flowering time, measured as leaves number at bolting (LNB), and CO log ratio of ZT8 over ZT15 expression in transgenic T1 lines for CO-A only (B) and both transgenes (C). The regression line is represented in dotted line surrounded by the confidence interval intervals (shaded area). (D) Comparison of the distribution of CO log-ratios between CO-A (orange) and CO-B (blue) T1s. (* = p < 0.05).
(PDF)

S1 Table A. arenosa site locations included in this study. (PDF)

S2 Table List of genes differentially expressed between RW and MT and most correlated (top 1%) with vernalization response (VR). (PDF)

S3 Table List of candidate introgressed genes with RW-like, MT-like expression profiles, or within top 5% outlier windows for G_ST_ between RW and MT. (DOCX)
