# Supplementary material for: Genetic basis and evolution of rapid cycling in railway populations of tetraploid Arabidopsis arenosa
Source: PLoS Genet. 2018 Jul 5;14(7):e1007510. doi: 10.1371/journal.pgen.1007510 (PMC6049958; doi:10.1371/journal.pgen.1007510)
Supplement: S1 Text — (PDF) [file pgen.1007510.s001.pdf]

Supplemental Text ST1.

Distribution of percentages of variance explained (PVE) across the *FLC*-*CO* region in TBG x SWA and BGS x KA.

To estimate individual effects of high LOD markers across the FT-peak region, we ran a step-wise multiple linear regression discarding markers that do not significantly improve the sum squared of error. We hereby obtained a reduced linear model relying on only 10 and 6 markers, but explaining 51.6% and 41.9% of the phenotypic variance observed in TBG x SWA and BGS x KA respectively. We then calculated the percentage of variance explained (PVE) by each of the 10 and 6 independent markers isolated using either a single-marker model (SMM) or the semi-partial correlation coefficients (SPC) of the multiple linear regression (Fig. S1C). The discrepancy between the two methods is due to the correlation between the markers that cannot be resolved by the power of our mapping scheme, leading to an overestimation of the PVEs in the SMM and an underestimation with the SPC approach. We were still able to establish a clear difference between the two crosses from the profiles of the SPC coefficients within the FT-QTL region: In TBG x SWA, the marker with the highest PVE fell within 75kb of *FLC*. In BGS x KA on the other hand, SNPs explaining some of the variance could be found within 75kb of *FLC*, but the highest specific PVE was at markers closer to *CO* (175 kb; Fig. S1C). This result suggests the QTL may be caused by *FLC* or a gene near it in TBG x SWA, but that in BGS x KA *FLC* may also contribute, but a gene at or near *CO* likely plays a larger role.

Patterson's *D*-statistic and modified *f*-statistics.

Both $\hat{f}_{hom}$ and $\hat{f}_d$ were calculated as described by Martin et al.[47]. using the frequency of derived alleles at each site in each population instead of binary counts:

$$(Equation\ 2) \qquad D(P_1,P_2,P_3,O) = \frac{\sum_{i=1}^{n}[(1-\widehat{P_{i1}})\widehat{P_{i2}}\widehat{P_{i3}}(1-\widehat{P_{iO}}) - \widehat{P_{i1}}(1-\widehat{P_{i2}})\widehat{P_{i3}}(1-\widehat{P_{iO}})]}{\sum_{i=1}^{n}[(1-\widehat{P_{i1}})\widehat{P_{i2}}\widehat{P_{i3}}(1-\widehat{P_{iO}}) + \widehat{P_{i1}}(1-\widehat{P_{i2}})\widehat{P_{i3}}(1-\widehat{P_{iO}})]}$$

where P_1, P_2, P_3 are the three populations used as background, receiver, and donor and O is *A. lyrata* (Fig. 3C). $\widehat{P_{ij}}$ are the observed frequencies of SNP i in population P_j. As we polarized alleles frequencies in our sample using a panel of 24 *A. lyrata* genomes, we simplified $Equation\ 2$ with $\widehat{P_{iO}}$=0. Similarly, we calculated the modified *f*-statistic $\hat{f}_{hom}$ described in Martin et al.[47] as:

$$(Equation\ 3) \qquad \hat{f}_{hom} = \frac{S(P_1,P_2,P_3,O)}{S(P_1,P_3,P_3,O)}$$

where $S(P_1,P_2,P_3,O)$ represents the numerator of $Equation\ 2$. We then split the genome into blocks of 50-kb which is greater than the very rapid LD decay observed in *A. arenosa* in order to avoid correlation between blocks. We used a leave-one-out jackknife approach on these blocks to evaluate the confidence intervals of our genome-wide estimates (D and $\hat{f}_{hom}$). To identify candidate introgression loci on a gene scale, we used the modified *f*-statistic $\hat{f}_d$ as:

$$(Equation\ 4) \qquad \hat{f}_d = \frac{S(P_1,P_2,P_3,O)}{S(P_1,P_D,P_D,O)}$$

where P_D is the highest value among P_2 and P_3 in order to take into account potential bi-directional gene-flow and incomplete lineage sorting which could give on a small window basis values of $\hat{f}_{hom}$ negative or above 1. We then calculated $\hat{f}_d$ for each gene when more than 25 informative SNPs were found across the gene annotation. We selected genes with high $\hat{f}_d$ values for each of the four railway-mountain couples ($\hat{f}_d(P_1,P_2,P_3) > 3 * \hat{f}_{hom}(P_1,P_2,P_3)$) in order to also take into account windows where introgression is high but introgressed alleles have not yet reached their equilibrium frequencies in BGS.
