# Supplementary material for: Genetic basis and evolution of rapid cycling in railway populations of tetraploid Arabidopsis arenosa
Source: PLoS Genet. 2018 Jul 5;14(7):e1007510. doi: 10.1371/journal.pgen.1007510 (PMC6049958; doi:10.1371/journal.pgen.1007510)
Supplement: S2 Text — (PDF) [file pgen.1007510.s002.pdf]

## Supplemental Text 2

We used our marker data in the FT QTL region (see Table ST2-4 below for marker locations) to infer chromosome complements of the F1 plants that were the parents of our F2 population. These plants are first generation progeny, and siblings, from a cross between a BGS plant and a KA plant. We color-coded the chromosomes for simplicity. Because the plants are tetraploids, each individual carries four chromosomes which sort two into each progeny.

The parent chromosomes in the regions are:

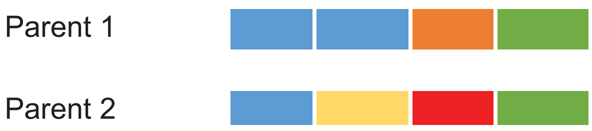


We then quantified, based on RADseq calls, how many of each chromosome each F2 plant must have in both the FLC and CO regions. We included further assessment only in individuals where we were able to make confident genotype calls. In different regions, these numbers varied as shown in Table ST2-1.

| | Chromosome | | | | |
|---|---|---|---|---|---|
| Gene region | Blue | Green | Orange | Red | Yellow |
| FLC | 135 | 121 | 87 | 101 | 87 |
| CO | 76 | 76 | 53 | 59 | 53 |

**Table ST2-1:** Number of individuals with high quality quantification by chromosome in each region

We then plotted how chromosome number correlated with the proportion of plants that successfully flowered before the end of the experiment (e.g. the proportion that were early; Figure ST1). The data are given below the figure in table form (Table ST2).

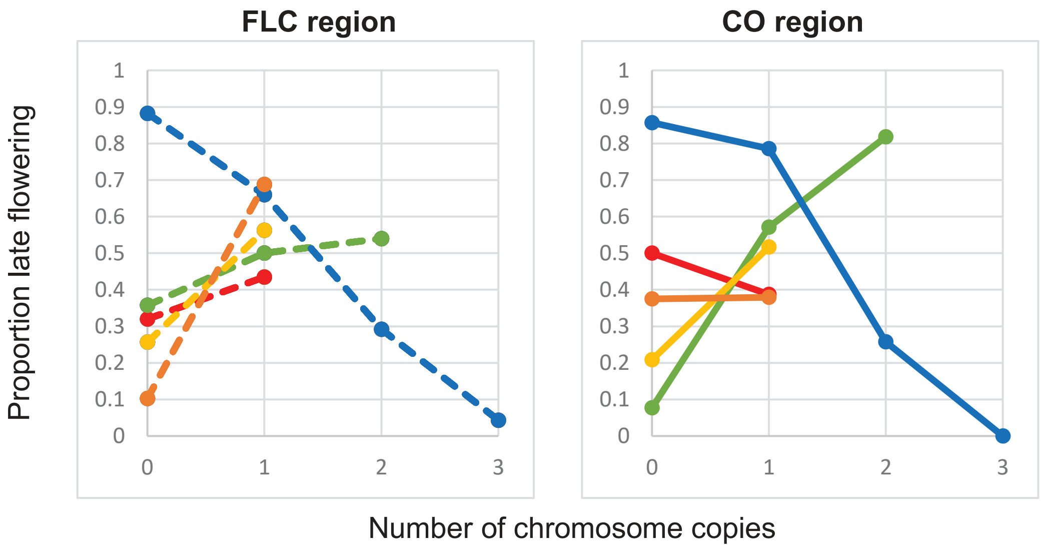


**Figure ST2-1:** Plots coded by chromosome color of the proportion of late flowering plants among individuals with the specified number of chromosome copies. Dotted line = effect at FLC, Solid line = effect at CO. Copy number is on X axis, proportion late is on the Y axis.

**FLC region**

| Proportion late by chromosome copy number | | | | Chromosome | Comments |
|---|---|---|---|---|---|
| 0 | 1 | 2 | 3 | | |
| 0.36 | 0.50 | 0.54 | | Green | Delays flowering (Dominant) |
| 0.88 | 0.66 | 0.29 | 0.04 | Blue | Accelerates flowering (Quantitative) |
| 0.26 | 0.56 | | | Yellow | Delays flowering |
| 0.32 | 0.43 | | | Red | Slight delay |
| 0.10 | 0.69 | | | Orange | Delays flowering |

**CO region**

| Proportion late by chromosome copy number | | | | Chromosome | Comments |
|---|---|---|---|---|---|
| 0 | 1 | 2 | 3 | | |
| 0.08 | 0.57 | 0.82 | | Green | Delays flowering (Quantitative) |
| 0.86 | 0.79 | 0.26 | 0.00 | Blue | Accelerates flowering (Quantitative) |
| 0.21 | 0.52 | | | Yellow | Delays flowering |
| 0.50 | 0.39 | | | Red | Slightly accelerates flowering |
| 0.38 | 0.38 | | | Orange | No effect |

**Table ST2-2:** Flowering effects of different chromosome copy numbers, given as proportion of late flowering plants. In each case, comments are given in the rightmost column.

Because the above analyses do not allow quantitative analysis because many plants did not flower, we also examined quantitative effects considering only plants that flowered before the end of the experiment (Table ST2 – 3). In each case, we plotted chromosomes against flowering time and report trends as $R^2$ values from linear regression.

| **Chromosome** | **Region** | **Trend** | **$R^2$** | **Conclusion** |
|---|---|---|---|---|
| Green | FLC | - | <0.001 | No effect |
| Green | CO | - | <0.01 | No effect |
| Blue | FLC | - | 0.01 | No effect |
| Blue | CO | Positive | **0.11** | Quantitative Acceleration |
| Yellow | FLC | - | 0.02 | No effect |
| Yellow | CO | (Negative) | 0.04 | Very weak repression |
| Red | FLC | (Positive) | 0.03 | Very weak acceleration |
| Red | CO | - | <0.01 | No effect |
| Orange | FLC | (Negative) | 0.02 | Very weak repression |
| Orange | CO | - | 0.01 | No effect |

**Table ST2-3.** Linear regression of chromosome copy numbers against flowering time, considering only plants that flowered before experiment end.

**Overall Summary:**
From the above data, we can say:
**Blue** chromosomes (BGS origin) – Quantitatively accelerate flowering. The effect is stronger at CO, and among early plants, is seen ONLY at CO.
**Green** – has repressive effects, also quantitative, especially at CO, it changes the proportion of late plants, but does not quantitatively affect early plants. The effect at FLC is weaker, but dominant.
**Yellow –** has repressive effects at both loci
**Orange** – has a repressive effect at FLC only

| Marker | Location (bp) | Comments |
|---|---|---|
| SNP2 | 4120220 | Nearest to FLC |
| SNP5 | 4180067 | |
| SNP6 | 4180073 | |
| SNP7 | 4907576 | |
| SNP8 | 5337954 | |
| SNP9 | 5355825 | |
| SNP10 | 5355834 | |
| SNP11 | 5750119 | |
| SNP12 | 5767206 | |
| SNP13 | 6291148 | |
| SNP14 | 6291166 | CO region |
| SNP15 | 6291208 | CO region |

**Table ST2-4:** Locations of markers used in FT-QTL region (Lyrata reference genome version 1; Hu et al. 2011).
