# Supplementary material for: Genetic basis and evolution of rapid cycling in railway populations of tetraploid Arabidopsis arenosa
Source: PLoS Genet. 2018 Jul 5;14(7):e1007510. doi: 10.1371/journal.pgen.1007510 (PMC6049958; doi:10.1371/journal.pgen.1007510)
Supplement: S1 Fig — (A) Volcano plots of differential expression (q-value) against log expression ratios between railway and mountain accessions (excluding BGS) within whole transcriptome. 5% most differentially expressed (two-tailed log-ratio) are highlighted in green and within these, flowering-time genes (FT) are in red. (B) Volcano plots of differential expression (q-value) against log expression ratios between railway and mountain accessions (excluding BGS) within FT-peak region. 5% most differentially expressed (two-tailed log-ratio) are highlighted in green and within these, flowering-time genes (FT) are in red. (C) Paralogue-specific FLC expression: relative expression of AaFLC1 (light grey) and AaFLC2 (dark grey) across mountain populations and BGS. (PDF) [file pgen.1007510.s003.pdf]

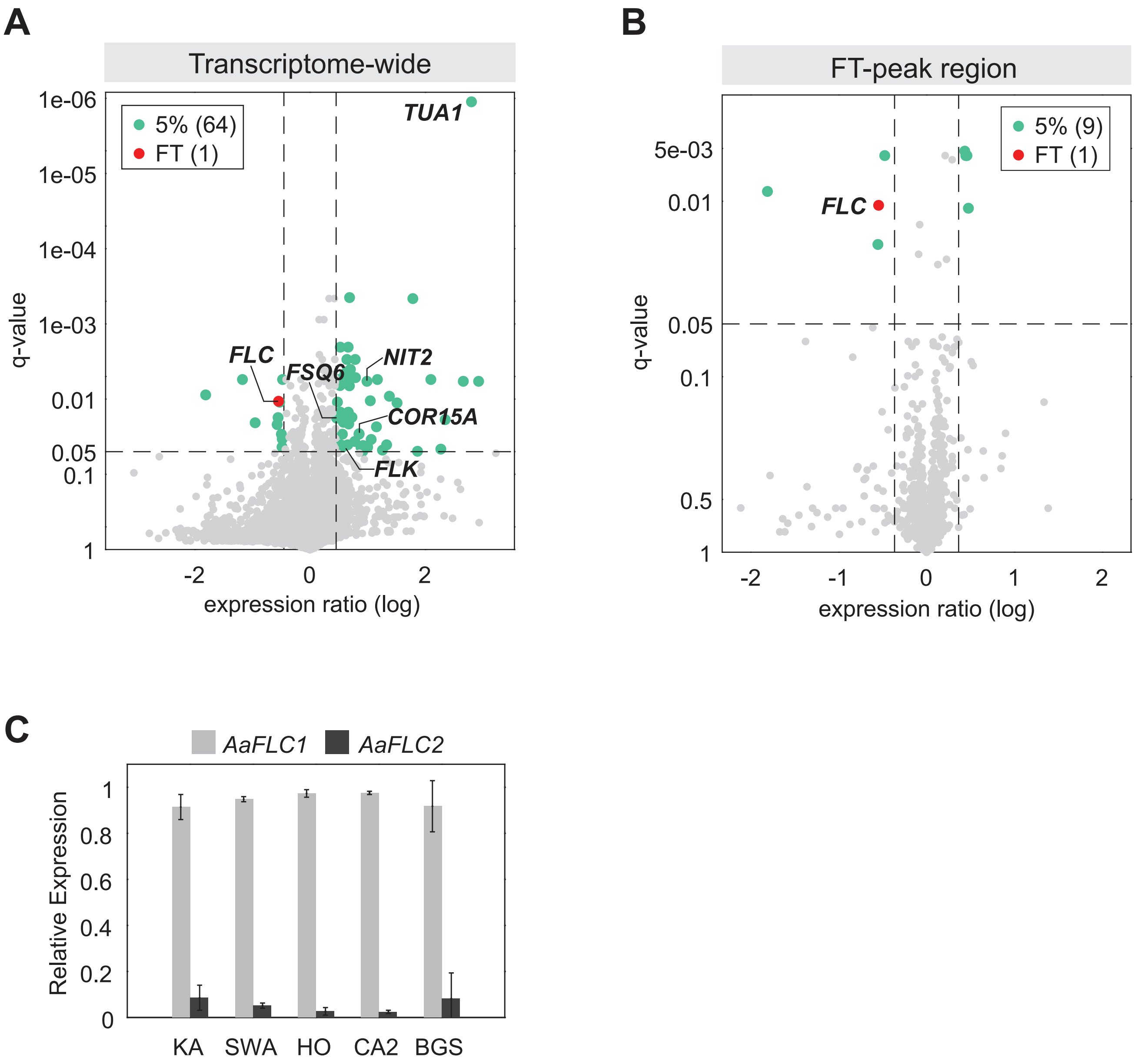

Figure S1. Differential expression between railway and mountain accessions.

(A) Volcano plots of differential expression (q-value) against log expression ratios between railway and mountain accessions (excluding BGS) within whole transcriptome. 5% most differentially expressed (two-tailed log-ratio) are highlighted in green and within these, flowering-time genes (FT) are in red. (B) Volcano plots of differential expression (q-value) against log expression ratios between railway and mountain accessions (excluding BGS) within FT-peak region. 5% most differentially expressed (two-tailed log-ratio) are highlighted in green and within these, flowering-time genes (FT) are in red. (C) Paralogue-specific *FLC* expression: relative expression of *AaFLC1* (light grey) and *AaFLC2* (dark grey) across mountain populations and BGS.
