# Supplementary material for: Genetic basis and evolution of rapid cycling in railway populations of tetraploid Arabidopsis arenosa
Source: PLoS Genet. 2018 Jul 5;14(7):e1007510. doi: 10.1371/journal.pgen.1007510 (PMC6049958; doi:10.1371/journal.pgen.1007510)
Supplement: S2 Fig — (A, B) Correlations between flowering time, measured as leaves number at bolting (LNB), and relative FLC expression in transgenic T1 lines for AaFLC1 and AaFLC2 35S-driven cDNA transgenes of KA (A) and BGS (B). The regression line is represented in dotted line surrounded by the confidence intervals (shaded area). Black triangles mark the two late-flowering individuals obtained with BGS AaFLC1 transgenes. Lines where transgene expression was below 50% of ACT expression (<0.5) are hollowed out. (PDF) [file pgen.1007510.s004.pdf]

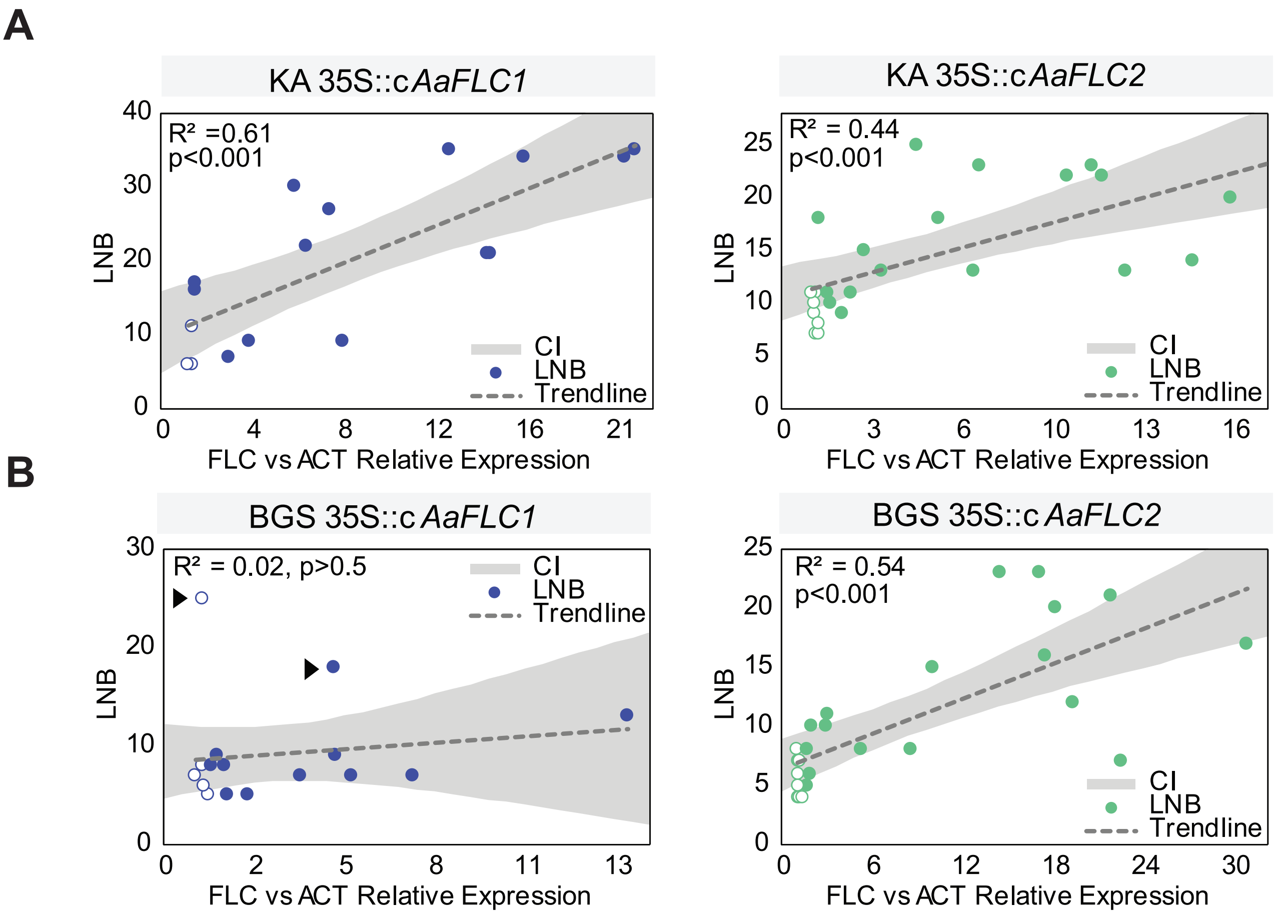

Figure S2. Correlation between Flowering Time and transgene expression.

(A, B) Correlations between flowering time, measured as leaves number at bolting (LNB), and relative *FLC* expression in transgenic T1 lines for *AaFLC1* and *AaFLC2* 35S-driven cDNA transgenes of KA (A) and BGS (B). The regression line is represented in dotted line surrounded by the confidence intervals (shaded area). Black triangles mark the two late-flowering individuals obtained with BGS *AaFLC1* transgenes. Lines where transgene expression was below 50% of ACT expression (<0.5) are hollowed out.
