# Supplementary material for: Genetic basis and evolution of rapid cycling in railway populations of tetraploid Arabidopsis arenosa
Source: PLoS Genet. 2018 Jul 5;14(7):e1007510. doi: 10.1371/journal.pgen.1007510 (PMC6049958; doi:10.1371/journal.pgen.1007510)
Supplement: S3 Fig — (A, B) Distribution of flowering time (Days to Bolting) in phenotyped (grey) and sequenced (blue) in TBG x SWA (A) and BGS x KA (B) F2 individuals. (C) Distribution of percentages of variance explained (PVE) across the FLC-CO region in TBG x SWA and BGS x KA. PVE distributions are shown for each cross above the gene models for the region. Single marker model (SMM) percent variance explained (PVE) are plotted in grey on the primary (left) y-axis, while semi-partial correlation coefficients (SPC) from the multiple linear model are in blue against the secondary y-axis. (PDF) [file pgen.1007510.s005.pdf]

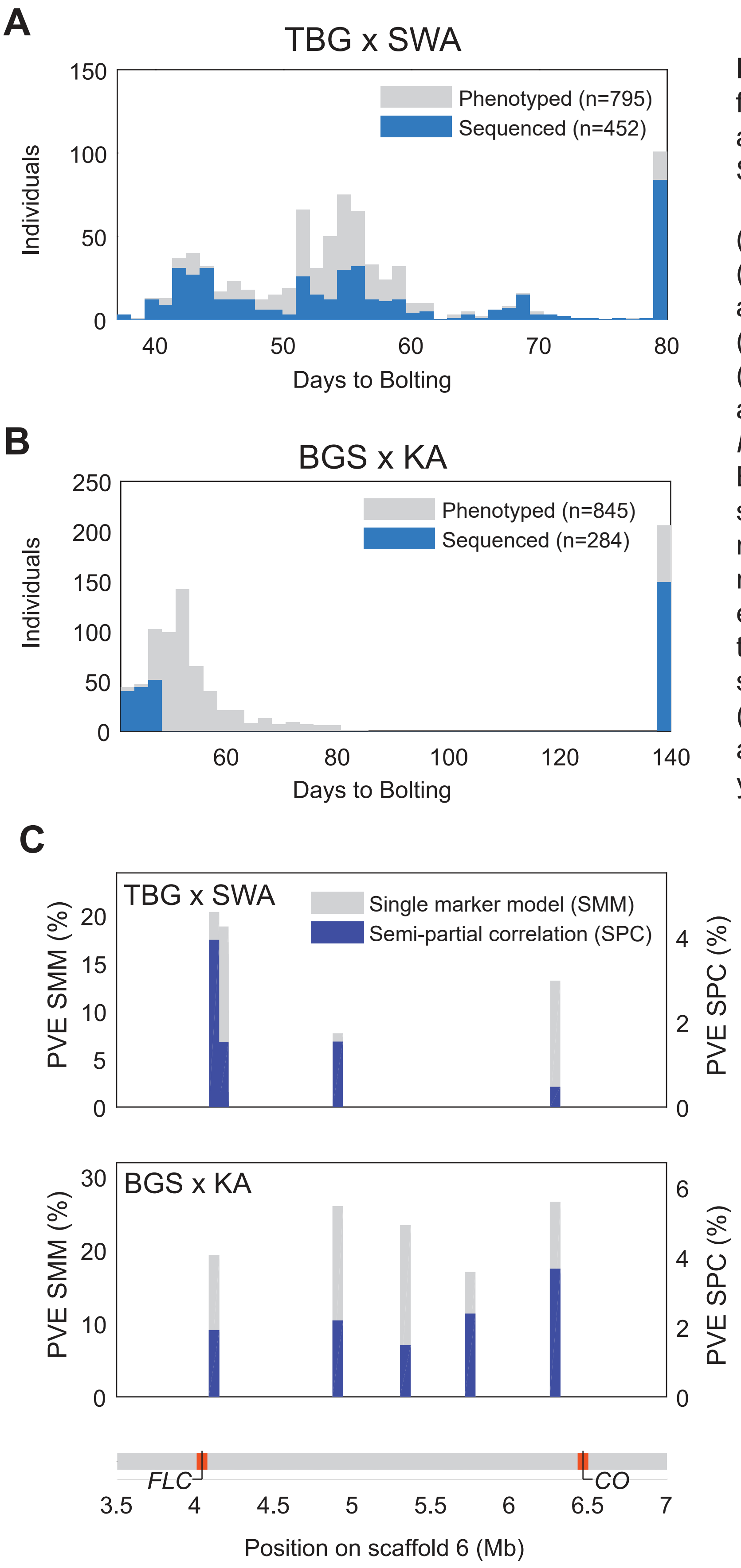

Figure S3. Phenotypic distribution of flowering time and PVE distribution across the *FLC-CO* region in TBG x SWA and BGS x KA F2s

(A, B) Distribution of flowering time (Days to Bolting) in phenotyped (grey) and sequenced (blue) in TBG x SWA (A) and BGS x KA (B) F2 individuals. (C) Distribution of percentages of variance explained (PVE) across the *FLC-CO* region in TBG x SWA and BGS x KA. PVE distributions are shown for each cross above the gene models for the region. Single marker model (SMM) percent variance explained (PVE) are plotted in grey on the primary (left) y-axis, while semi-partial correlation coefficients (SPC) from the multiple linear model are in blue against the secondary y-axis.
