# Supplementary figures and images for: Genetic basis and evolution of rapid cycling in railway populations of tetraploid Arabidopsis arenosa
Source: PLoS Genet. 2018 Jul 5;14(7):e1007510. doi: 10.1371/journal.pgen.1007510 (PMC6049958; doi:10.1371/journal.pgen.1007510)

**A**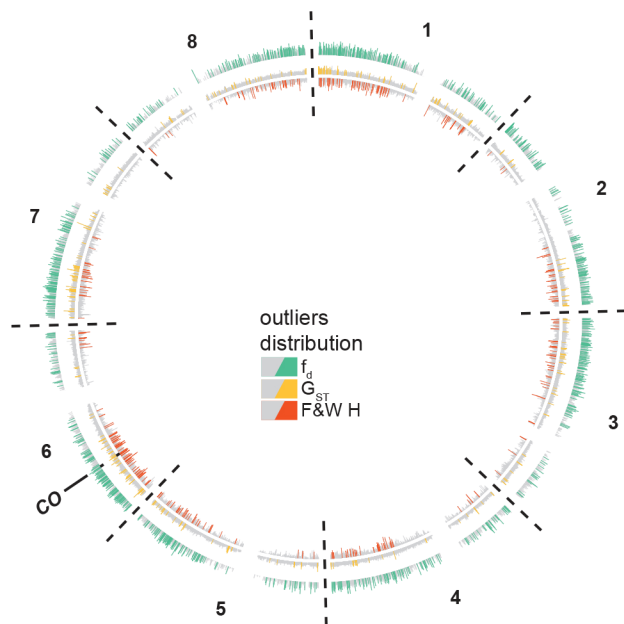**B**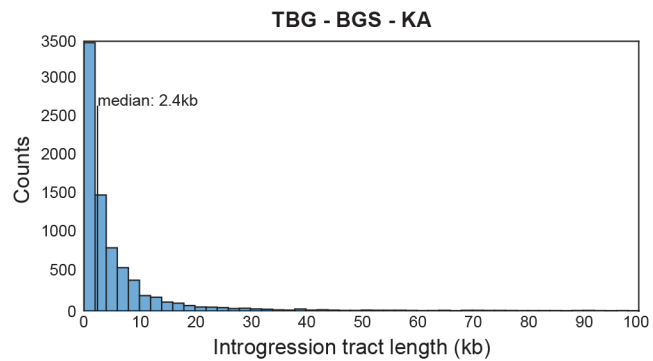**C**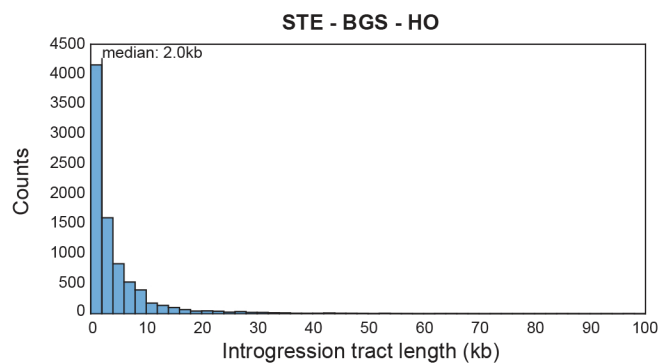**D**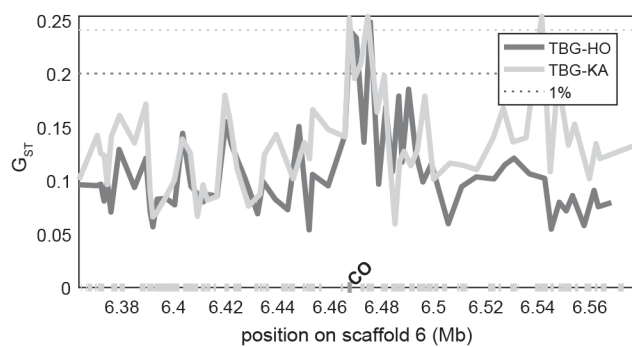**E**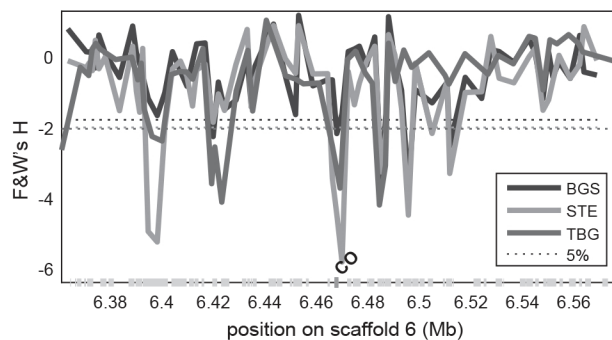

Supplement: S4 Fig — (A) Distribution of whole-genome (grey) and outliers values of mean fd across all 4 RW-MT pairs (green, outer ring), mean GST across all 4 RW-MT pairs (yellow, middle ring). (B,C) Distribution of introgression tract lengths for TBG-BGS-KA and STE-BGS-HO respectively. (D) Marks of differentiation between one railway population (TBG) and two mountain populations (HO and KA) evaluated with GST across CO region. Dotted lines are respective genome-wide 1% threshold levels. (E) Fay and Wu’s H on 200kb region surrounding CO, with genome-wide 5% threshold levels (dotted lines). (PDF) [file pgen.1007510.s006.pdf]
