# Supplementary material for: Genetic basis and evolution of rapid cycling in railway populations of tetraploid Arabidopsis arenosa
Source: PLoS Genet. 2018 Jul 5;14(7):e1007510. doi: 10.1371/journal.pgen.1007510 (PMC6049958; doi:10.1371/journal.pgen.1007510)
Supplement: S5 Fig — Map showing the rail network in Germany and surrounding areas from 1849. The railways are indicated as solid bold black lines. Lines added by 1861 are shown as dotted lines illustrating the rapid expansion of a widely connected transport network. Map image is public domain and obtained from Wikipedia: https://en.wikipedia.org/wiki/History_of_rail_transport_in_Germany. (PDF) [file pgen.1007510.s007.pdf]

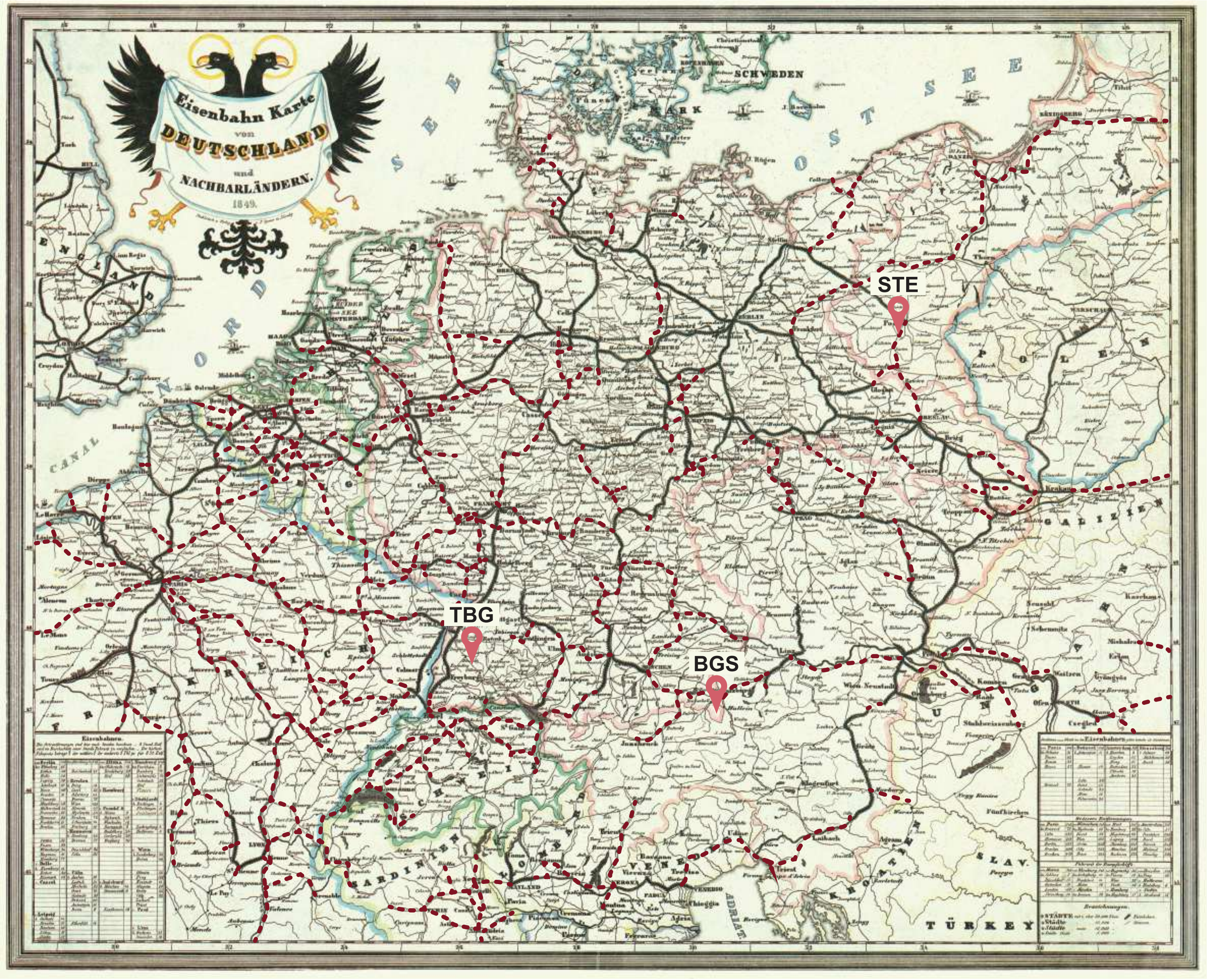

Figure S5. Rail networks in central Europe from 1849-1861.

Map showing the rail network in Germany and surrounding areas from 1849. The railways are indicated as solid bold black lines. Lines added by 1861 are shown as dotted lines illustrating the rapid expansion of a widely connected transport network. Map image is public domain and obtained from Wikipedia: https://en.wikipedia.org/wiki/History_of_rail_transport_in_Germany.
