# Supplementary material for: Genetic basis and evolution of rapid cycling in railway populations of tetraploid Arabidopsis arenosa
Source: PLoS Genet. 2018 Jul 5;14(7):e1007510. doi: 10.1371/journal.pgen.1007510 (PMC6049958; doi:10.1371/journal.pgen.1007510)
Supplement: S6 Fig — (A) Normalized CO expression at ZT15 of CO-A (orange) and CO-B (blue) T1s. (B-C) Correlations between flowering time, measured as leaves number at bolting (LNB), and CO log ratio of ZT8 over ZT15 expression in transgenic T1 lines for CO-A only (B) and both transgenes (C). The regression line is represented in dotted line surrounded by the confidence interval intervals (shaded area). (D) Comparison of the distribution of CO log-ratios between CO-A (orange) and CO-B (blue) T1s. (* = p < 0.05). (PDF) [file pgen.1007510.s008.pdf]

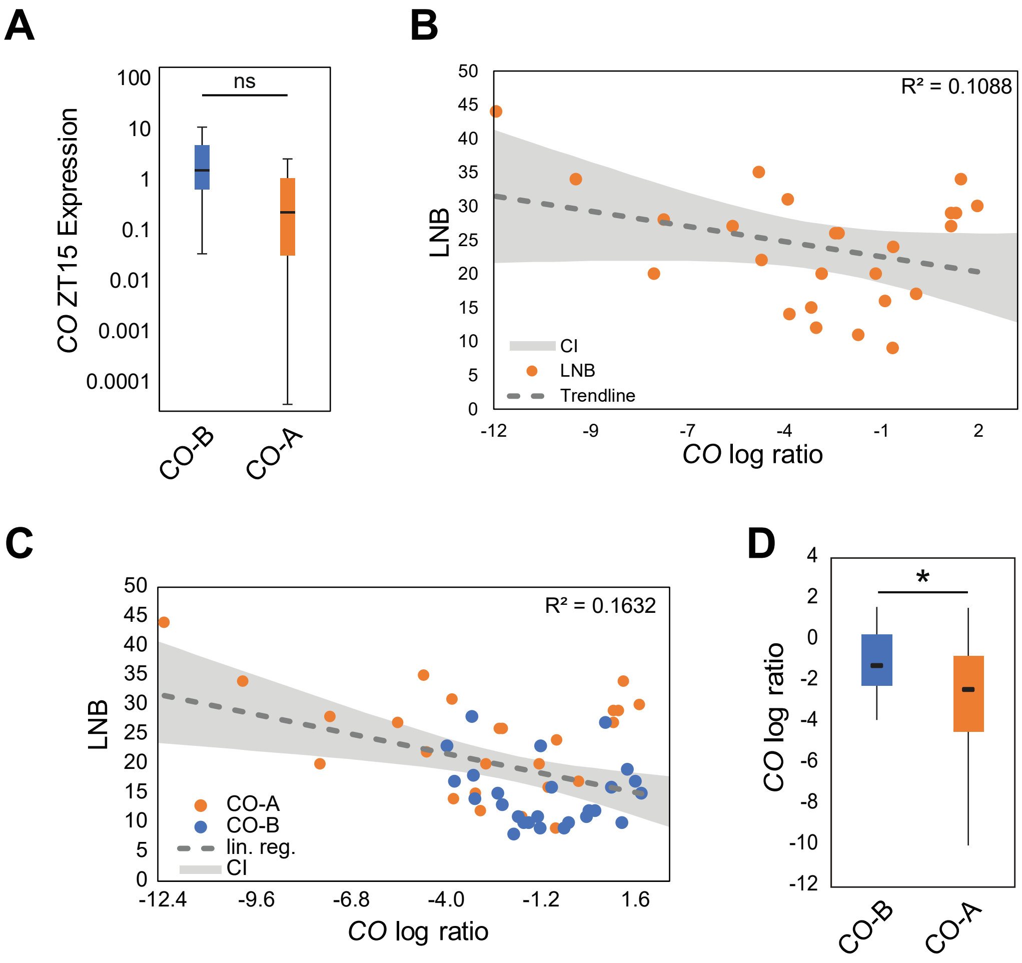

Figure S6. *CO* expression in *CO-A* and *CO-B* transgenic lines.

(A) Normalized *CO* expression at ZT15 of *CO-A* (orange) and *CO-B* (blue) T1s. (B-C) Correlations between flowering time, measured as leaves number at bolting (LNB), and *CO* log ratio of ZT8 over ZT15 expression in transgenic T1 lines for *CO-A* only (B) and both transgenes (C). The regression line is represented in dotted line surrounded by the confidence intervals (shaded area). (D) Comparison of the distribution of *CO* log-ratios between *CO-A* (orange) and *CO-B* (blue) T1s. (* = $p < 0.05$)
