# Supplementary material for: Genetic basis and evolution of rapid cycling in railway populations of tetraploid Arabidopsis arenosa
Source: PLoS Genet. 2018 Jul 5;14(7):e1007510. doi: 10.1371/journal.pgen.1007510 (PMC6049958; doi:10.1371/journal.pgen.1007510)
Supplement: S1 Table — (PDF) [file pgen.1007510.s009.pdf]

Table S1: *A. arenosa* site locations included in this study

Tetraploid transcriptomes

Abbreviation	Site location	GPS - Longitude	GPS - Latitude	Altitude (m)	Site type
STE	Stęszew, Poland	52° 16' 49" N	16° 42' 34" E	78	Railway ballast / platforms
TBG	Triberg, Germany	48° 8' 24.7" N	8° 14' 13.2" E	638	Railway ballast / platforms
BGS	Berchtesgaden, Germany	47° 37' 41" N	13° 0' 06" E	552	Railway ballast / platforms
SWA	Upfinger Steige, Bad Urach, Germany	48° 28' 32.6" N	9° 23' 47.5" E	739	Hill region, Shaded limestone outcrop
HO	Hochlantsch,Mautstatt, Austria	47° 22' 12" N	15° 23' 12" E	533	Mountain region, Shaded outcrop
CA2	Hnilčík, Slovakia	48° 52' 21" N	20° 31' 30" E	841	Mountain region, Shaded embankment
KA	Kasparstein, Austria	46° 41' 18" N	14° 52' 18" E	660	Mountain region, Limestone outcrops / Castle ruin

172 diploid and tetraploid genomes

Abbreviation	Ploidy	GPS - Longitude	GPS - Latitude	Individuals	Clade
MIE	2x	14° 25' 18" N	53° 55' 16" E	8	Baltic
PRE	2x	21° 1' 56" N	55° 22' 42" E	8	Baltic
FOJ	2x	17° 49' 28" N	43° 58' 30" E	7	Dinaric
BIH	2x	15° 53' 56" N	44° 52' 55" E	8	Dinaric
SZI	2x	17° 26' 4" N	46° 48' 24" E	5	Pannonian
HNE	2x	19° 0' 0" N	48° 16' 1" E	7	Pannonian
KZL	2x	18° 46' 45" N	47° 43' 28" E	5	Pannonian
RZA	2x	22° 45' 30" N	45° 22' 40" E	9	S. carp.
GOR	2x	21° 32' 34" N	44° 15' 55" E	8	S. carp.
SNO	2x	18° 51' 42" N	49° 10' 27" E	6	W. carp.
VEL	2x	20° 9' 15" N	49° 9' 43" E	8	W. carp.
TRD	2x	20° 12' 23" N	49° 15' 6" E	6	W. carp.
TKO	4x	19° 44' 7" N	49° 12' 16" E	8	W. carp.
SPI	4x	20° 46' 30" N	48° 59' 20" E	13	W. carp.

TZI	4x	23° 40' 27" N	46° 34' 0" E	9	S. carp.
DRA	4x	25° 13' 26" N	45° 26' 30" E	7	S. carp.
SCH	4x	14° 19' 19" N	47° 16' 40" E	7	Alps
HOC	4x	15° 23' 12" N	47° 22' 12" E	6	Alps
KAS	4x	14° 52' 18" N	46° 41' 18" E	8	Alps
STE	4x	16° 42' 34" N	52° 16' 49" E	8	railway
KOW	4x	15° 50' 38" N	50° 45' 47" E	8	railway
TBG	4x	8° 14' 12" N	48° 8' 23" E	5	railway
BGS	4x	13° 0' 6" N	47° 37' 41" E	8	railway/alps
