# Supplementary material for: Genetic basis and evolution of rapid cycling in railway populations of tetraploid Arabidopsis arenosa
Source: PLoS Genet. 2018 Jul 5;14(7):e1007510. doi: 10.1371/journal.pgen.1007510 (PMC6049958; doi:10.1371/journal.pgen.1007510)
Supplement: S2 Table — (PDF) [file pgen.1007510.s010.pdf]

Table S2: List of genes differentially expressed between RW and MT and most correlated (top 1%) with vernalization response (VR)

Correlations with vernalization response (VR) exclude BGS

				TBG	BGS	STE	CA2	HO	US	KA
**Vernalization Response (days)**				6.1	8.5	17.3	45.0	76.1	105.0	124.3
***A. lyrata* ID**	***A. thaliana* ID**	**Alias**	**Outlier in BGS at 5%**	**Population Mean Expression (normalized gene counts)**						
314357	AT3G46220		*yes*	15.1	65.6	23.8	12.5	72.6	111.3	165.9
320642	AT2G28540		*yes*	335.0	422.4	342.9	363.9	362.7	391.3	420.7
322004	AT2G43270		*yes*	31.3	7.7	28.6	19.4	17.9	1.1	1.0
329536	AT4G20350		*yes*	46.5	22.8	50.5	39.2	40.4	28.8	23.2
342776	AT2G04360		*no*	272.1	314.8	301.0	339.5	338.4	390.6	402.1
344203			*yes*	0.0	9.9	1.0	5.2	12.7	12.6	23.2
348353	AT3G52160	*KCS15*	*yes*	49.2	0.0	37.6	34.9	13.9	0.4	0.0
356659	AT5G44240	*ALA2*	*yes*	281.9	328.3	316.1	328.8	350.2	365.3	367.7
356912	AT5G42350	*CFK1*	*yes*	52.6	76.3	56.1	60.4	70.1	72.7	71.1
470299	AT1G03190	*UVH6*	*yes*	202.8	174.3	190.2	172.9	173.6	164.6	155.8
472974	AT1G27695		*yes*	98.8	173.6	129.5	121.7	142.2	153.9	160.0
473646	AT1G34760	*GRF11*	*yes*	33.5	77.7	74.0	59.4	140.4	130.9	188.9
473746	AT1G42960		*yes*	619.5	729.9	632.0	690.8	735.7	718.1	829.0

475117	AT1G62290		*yes*	112.8	400.1	184.9	232.6	340.9	418.5	384.4
475676	AT1G65720		*yes*	558.7	670.8	551.6	589.2	637.0	646.8	657.8
475678	AT3G24550	*PERK1*	*yes*	14.5	83.4	19.7	26.2	29.3	30.2	31.4
477520	AT3G02780	*IPIAT1*	*yes*	405.7	506.4	449.1	452.7	485.3	542.8	525.2
477930	AT3G06310		*yes*	159.4	179.4	160.4	163.1	199.0	209.0	212.8
478302	AT3G09790	*UBQ8*	*yes*	28.4	14.7	26.4	18.3	17.2	16.3	10.0
478575	AT3G12150		*yes*	125.5	164.7	122.8	155.8	162.2	171.3	215.3
479233	AT3G17570		*no*	14.1	16.3	22.0	25.6	34.5	40.5	40.5
481399	AT2G25480		*yes*	463.2	385.3	437.4	387.0	356.9	368.4	319.8
481510	AT2G26510	*PDE135*	*yes*	347.1	385.7	371.6	377.8	403.8	396.7	417.0
481514	AT2G26540	*ATUROS*	*no*	444.6	499.2	488.6	518.3	513.2	565.5	625.7
482268	AT5G20620	*UBQ4*	*yes*	20.4	82.4	44.4	50.1	114.1	124.2	108.5
482579	AT2G36050	*OFP15*	*yes*	114.8	54.9	88.6	95.2	70.2	63.7	51.5
482729	AT2G37360	*ABCG2*	*no*	121.0	115.8	123.9	112.7	102.8	102.5	99.3
483408	AT2G43060	*IBH1*	*no*	28.2	23.2	25.3	27.4	9.1	8.6	7.8
483513	AT2G43950	*OEP37*	*yes*	278.9	384.5	320.7	347.9	387.2	397.0	438.8
483654	AT2G45180		*yes*	484.1	945.8	378.6	601.4	801.5	847.1	984.1
484689	AT3G29200	*CM1*	*yes*	341.5	263.9	316.1	306.3	288.0	269.6	247.9
486208	AT3G57590		*yes*	31.7	11.3	24.1	23.2	15.3	15.6	9.1
486946	AT5G01310	*APTX*	*yes*	87.3	65.9	80.7	73.6	57.5	65.3	53.1
487083	AT5G03120		*yes*	111.1	167.4	116.5	151.9	149.3	161.2	197.0
487412	AT5G06100	*MYB33*	*yes*	43.8	57.2	48.9	51.2	62.0	64.6	70.7

488468	AT5G16160		*yes*	18.8	12.7	16.2	14.8	11.3	9.1	8.9
488687	AT5G18200		*yes*	195.8	340.7	171.7	226.5	262.9	280.4	347.9
489200	AT5G23390		*yes*	270.4	339.2	265.7	324.9	339.7	362.0	374.9
489875	AT4G10320		*yes*	1053.4	981.1	1081.0	944.3	882.1	852.6	813.5
490081	AT4G04220	*RLP46*	*yes*	17.1	68.5	42.1	56.2	69.9	139.6	118.4
490426	AT4G01480	*PPa5*	*yes*	72.3	127.4	73.6	102.0	95.0	152.5	198.1
490517	AT4G00490	*BMY9*	*no*	696.0	603.1	567.8	531.5	516.2	477.6	325.0
490805	AT4G38080		*yes*	23.0	10.6	21.8	18.2	14.3	13.0	6.0
490998	AT4G36390		*yes*	913.0	1037.3	970.0	1071.6	1097.0	1111.1	1138.9
491814	AT4G29440		*yes*	327.2	247.8	340.4	266.0	286.6	226.3	191.6
494338	AT5G46340	*RWA1*	*yes*	165.8	126.2	163.9	133.1	117.7	123.1	80.2
495057	AT5G50010		*yes*	13.2	6.3	10.8	9.7	8.0	5.7	4.8
496494	AT5G63120		*yes*	265.6	209.6	286.6	197.1	171.2	179.4	128.2
888289	AT2G44270	*ROL5*	*no*	7.9	8.7	5.5	4.3	4.2	0.8	1.4
889149	AT4G29760		*no*	51.4	39.1	39.6	28.1	22.4	20.1	11.6
893821	AT5G19030		*yes*	93.8	64.0	90.1	73.4	67.7	59.6	31.5
897389	AT5G60050		*yes*	15.8	6.6	15.2	8.2	0.0	0.0	0.0
899731			*yes*	16.9	0.0	14.3	7.8	2.3	1.2	0.0
902358	AT5G55160	*SUM2*	*no*	46.6	43.8	52.6	40.1	23.6	10.1	10.0
904299	AT3G06530		*no*	72.5	52.6	42.8	50.3	25.2	0.4	7.3
904726	AT3G17620		*yes*	0.6	47.6	7.2	0.0	42.8	40.0	80.0
908155	AT1G66520	*pde194*	*no*	4.0	4.9	2.3	6.1	8.3	12.1	13.0

909580	AT1G79500	*AtkdsA1*	*yes*	153.9	219.8	174.9	213.9	220.6	251.2	258.2
911846	AT1G20150		*yes*	0.0	14.5	0.4	2.3	8.7	9.9	15.4
919118	AT1G04420		*yes*	706.2	964.0	685.2	851.9	881.9	918.6	957.2
920544			*no*	0.6	1.9	0.0	1.3	5.7	4.2	9.0
923384	AT1G49630	*PREP2*	*yes*	362.4	225.2	323.6	307.2	233.8	156.0	196.2
923799	AT3G15640		*no*	88.2	136.3	134.8	133.5	161.2	155.7	214.6
924987	AT1G60670		*yes*	110.9	72.2	117.6	109.0	97.8	81.9	87.0
926001	AT1G78960	*LUP2*	*yes*	31.5	2.8	22.2	17.8	1.8	2.7	0.6
928125	AT3G05350		*no*	497.3	579.3	520.5	620.0	699.4	676.4	717.2
928349	AT3G06910	*ELS1*	*yes*	34.2	43.0	32.5	27.5	25.4	26.8	22.2
928634	AT5G41820	*RGTA2*	*yes*	7.0	43.9	4.3	11.8	54.4	43.9	69.9
931581			*yes*	79.3	7.3	79.9	76.1	24.3	33.8	18.4
939212	AT3G61580	*SLD1*	*yes*	613.0	773.1	720.7	634.3	833.8	884.7	904.2
940484	AT5G10140	*FLC*	*yes*	37.1	164.5	46.6	119.3	126.4	139.2	203.9
943375	AT4G03400	*GH3-10*	*no*	664.4	808.4	711.0	890.3	864.7	1064.4	1050.4
945513	AT1G64990	*GTG1*	*no*	3.7	3.8	1.6	4.9	7.7	9.3	8.8
947253	AT4G00895		*no*	13.0	14.5	12.3	17.5	20.2	33.5	31.1
950227	AT5G55710	*Tic20-V*	*yes*	324.7	392.0	376.1	416.4	418.2	441.9	484.5
952522	AT3G13640	*ATRLI1*	*no*	1.3	3.9	3.7	3.3	8.2	7.5	8.6
