# Supplementary material for: Genetic basis and evolution of rapid cycling in railway populations of tetraploid Arabidopsis arenosa
Source: PLoS Genet. 2018 Jul 5;14(7):e1007510. doi: 10.1371/journal.pgen.1007510 (PMC6049958; doi:10.1371/journal.pgen.1007510)
Supplement: S3 Table — (DOCX) [file pgen.1007510.s011.docx]

| **Table S3: List of candidate introgressed genes with RW-like, MT-like expression profiles, or within top 5% outlier windows for Fay & Wu's H in lowland Railways (RW) and G_ST_ between RW and MT** | | | | | | | | | |
| --- | --- | --- | --- | --- | --- | --- | --- | --- | --- |
|  |  |  |  |  |  |  |  | **Fay & Wu’s H + G_ST_(RW/MT) outliers (5%)** | |
| **Railway-like** | | |  | **Mountain-like** | | |  |  |  |
| ***A. lyrata* ID** | ***A. thaliana* ID** | **Alias** |  | ***A. lyrata* ID** | ***A. thaliana* ID** | **Alias** |  | ***A. thaliana* ID** | **Alias** |
| 478894 | AT3G14810 | *MSL5* |  | 323329 | AT3G47010 |  |  | AT1G18390 |  |
| 484639 | AT3G28640 |  |  | 474176 | AT1G50480 | *THFS* |  | AT2G41000 |  |
| 485462 | AT3G51090 |  |  | 479964 | AT3G24520 | *HSFC1* |  | AT5G55300 | *TOP1* |
| 902358 | AT5G55160 | *SUM2* |  | 483719 | AT2G45810 |  |  | AT1G63010 |  |
| 921642 | AT1G26180 |  |  | 907722 | AT1G64820 |  |  | AT1G61170 |  |
| 944648 | AT4G32970 |  |  | 927567 | AT3G01890 |  |  | AT1G61140 | *EDA16* |
|  |  |  |  | 929526 | AT3G16860 | *COBL8* |  | AT5G13960 | *SDG33* |
|  |  |  |  | 940349 | AT5G08565 |  |  | AT3G46610 |  |
|  |  |  |  | 945215 | AT4G28200 |  |  | AT5G15840 | *CO* |
|  |  |  |  |  |  |  |  | AT4G01870 |  |
|  |  |  |  |  |  |  |  | AT4G25960 | *PGP2* |
|  |  |  |  |  |  |  |  | AT3G10440 |  |
|  |  |  |  |  |  |  |  | AT1G03120 | *RAB28* |
|  |  |  |  |  |  |  |  | AT1G03130 | *PSAD-2* |
|  |  |  |  |  |  |  |  | AT1G09010 |  |
|  |  |  |  |  |  |  |  | AT3G17360 | *POK1* |
|  |  |  |  |  |  |  |  | AT1G04430 |  |
|  |  |  |  |  |  |  |  | AT4G26710 |  |
|  |  |  |  |  |  |  |  | AT5G50210 | *QS* |
